# Using a Digital Twin of an Electrical Stimulation Device to Monitor and Control the Electrical Stimulation of Cells *in vitro*

**DOI:** 10.3389/fbioe.2021.765516

**Published:** 2021-12-08

**Authors:** Julius Zimmermann, Kai Budde, Nils Arbeiter, Francia Molina, Alexander Storch, Adelinde M. Uhrmacher, Ursula van Rienen

**Affiliations:** ^1^ Institute of General Electrical Engineering, University of Rostock, Rostock, Germany; ^2^ Institute for Visual and Analytic Computing, University of Rostock, Rostock, Germany; ^3^ Department of Neurology, University of Rostock, Rostock, Germany; ^4^ Department Life, Light and Matter, University of Rostock, Rostock, Germany; ^5^ Department Ageing of Individuals and Society, University of Rostock, Rostock, Germany

**Keywords:** electrical stimulation, in silico modeling, uncertainty quantification, electrochemical impedance spectroscopy, cell culture experiments, deep brain stimulation, tissue engineering, regenerative medicine

## Abstract

Electrical stimulation for application in tissue engineering and regenerative medicine has received increasing attention in recent years. A variety of stimulation methods, waveforms and amplitudes have been studied. However, a clear choice of optimal stimulation parameters is still not available and is complicated by ambiguous reporting standards. In order to understand underlying cellular mechanisms affected by the electrical stimulation, the knowledge of the actual prevailing field strength or current density is required. Here, we present a comprehensive digital representation, a digital twin, of a basic electrical stimulation device for the electrical stimulation of cells *in vitro*. The effect of electrochemical processes at the electrode surface was experimentally characterised and integrated into a numerical model of the electrical stimulation. Uncertainty quantification techniques were used to identify the influence of model uncertainties on relevant observables. Different stimulation protocols were compared and it was assessed if the information contained in the monitored stimulation pulses could be related to the stimulation model. We found that our approach permits to model and simulate the recorded rectangular waveforms such that local electric field strengths become accessible. Moreover, we could predict stimulation voltages and currents reliably. This enabled us to define a controlled stimulation setting and to identify significant temperature changes of the cell culture in the monitored voltage data. Eventually, we give an outlook on how the presented methods can be applied in more complex situations such as the stimulation of hydrogels or tissue *in vivo*.

## 1 Introduction

In recent years, electrical stimulation has (re-)emerged as a possible tool for tissue engineering and regenerative medicine (Balint et al., 2013; da Silva et al., 2020). Applications include wound healing (Zhao et al., 2006; Zhao, 2009), cardiac tissue engineering (Tandon et al., 2009), neural stimulation (Iwasa et al., 2020), bone regeneration (Meng et al., 2013), and cartilage regeneration (Jahr et al., 2015). As a prominent example, deep brain stimulation (DBS) has been established as a clinical therapy (Krauss et al., 2020) with already more than 160,000 patients treated (Lozano et al., 2019). However, the underlying processes at the cellular and molecular scales are not well understood. Hence, *in vitro* experiments have been designed to elucidate possible mechanisms of cellular response to electrical stimulation (Zhao et al., 2020). The ever-growing number of publications on *in vitro* electrical stimulation has been covered in a considerable amount of literature reviews (Funk et al., 2009; Balint et al., 2013; Jahr et al., 2015; Thrivikraman et al., 2018; Chen et al., 2019; da Silva et al., 2020; Ryan et al., 2021).

Thanks to advanced instrumentation, many parameters can be considered in electrical stimulation; these are, amongst others, waveform, amplitude and frequency of the stimulation signal, electrode material and the method of delivering the stimulation. In this work, we focus on the direct contact electrical stimulation where the electrodes are placed in an electrolyte (e.g., cells in medium). In this approach, the delivery of the stimulation signal is closely related to electrochemical processes (Richardot and McAdams, 2002). Because the byproducts of electrochemical stimulation might be harmful, safe stimulation parameters and electrode materials need to be chosen (Merrill et al., 2005; Boehler et al., 2020). Noble metals (platinum, gold, iridium and corresponding oxides/alloys) have emerged as popular electrode materials due to their corrosion resistance. Furthermore, safe waveforms have been identified (Merrill et al., 2005). In DBS, for example, charge-balanced rectangular pulses are frequently used (Krauss et al., 2020). In this respect, novel stimulation paradigms have been suggested; for example, the use of (high frequency) kilohertz stimulation (Neudorfer et al., 2021). The abundance of stimulation parameters and external influences on the stimulation makes it necessary to develop a solid understanding of the effect of novel electrical stimulation approaches. For that, realistic *in vitro* models are studied with the goal of translation into *in vivo* applications.

With the rise of sophisticated numerical tools, these efforts can be accompanied by *in silico* modelling. Ideally, a digital twin (i.e., a digital representation) of the electrical stimulation experiment can be established. Following the argumentation of Wright and Davidson (2020), a digital twin consists of three parts, which we will address in this work: “a model of the object, an evolving set of data relating to the object, and a means of dynamically updating or adjusting the model in accordance with the data” (Wright and Davidson, 2020). Having a reliable digital twin at hand helps to make optimal experiment choices and save time and resources on the way to an improved *in vitro* outcome (Geris et al., 2018). Even quantities that are difficult to measure or cannot even be measured are then accessible. In the context of electrical stimulation, a digital twin should facilitate the choice of the stimulation parameters and eventually contribute to the explanation of the observed biological response. Hence, it should also possess predictive power. Then, a digital twin can serve also as a tool for performance assurance (i.e., as an indicator for undesired or unexpected processes or even failure of the stimulation approach). A challenge for accurate modelling is the electrode-electrolyte interface (EEI) where possibly non-linear electrochemical reactions occur (Richardot and McAdams, 2002). Evidently, a digital twin of the stimulation experiment has to account for the electrochemical processes at the EEI. Usually, the EEI has been modelled based on prior knowledge (Cantrell et al., 2008). Here, we want to explore if this approach can be improved using data that is collected *in situ*. Recently, a dual-function apparatus for *in vitro* electrical stimulation has been presented (Abasi et al., 2020). It monitors the electrochemical status of the cell culture when no electrical stimulation is actively applied by impedance spectroscopy. In this work, we aim at deriving impedance spectra from the recorded stimulation pulses and thus at gaining information about the system while it is actively stimulated.

Numerical models are often approached with scepticism regarding their validity. Particularly in biosciences, verification, validation and uncertainty quantification (VVUQ) become highly important (Coveney and Highfield, 2021). In the context of electrical stimulation, the need for thorough VVUQ is corroborated by problems in the reproducibility of experimental studies, which have been identified in recent research (Portelli et al., 2018; Budde et al., 2019; Guette-Marquet et al., 2021). Because building a validated model requires detailed information about the experiment, it can contribute to improved documentation and thus its reproducibility. Strikingly, the reported electric field strengths appear to be highly uncertain. It is an important parameter for the characterisation and quantification of *in vitro* electrical stimulation experiments and often used for comparison (Thrivikraman et al., 2018; Ryan et al., 2021). Unfortunately, the field strength cannot be measured directly, but only be inferred from other measurement results (Gundersen and Greenebaum, 1985). A non-invasive approach to estimate the field strength relies on the measurement of the current through the sample. Hence, it has been advocated to report this quantity (Schopf et al., 2016; Guette-Marquet et al., 2021). Additional reporting standards and guidelines have been suggested for direct current stimulation (Tandon et al., 2009), electrochemical tests of stimulating electrodes (Boehler et al., 2020) and *in vitro* low-frequency electromagnetic stimulation (Misakian et al., 1993). Nevertheless, clear standards for numerical simulations and the respective VVUQ for electrical stimulation applications are still lacking.

In this work, we address the aforementioned reproducibility problem and establish a connection between theory and experiment to highlight, which information needs to be provided for improved reproducibility regarding the applied electrical field strengths and currents. We will not address the biological aspects of electrical stimulation. We study electrical stimulation with direct current as well as rectangular signals using an *in vitro* stimulation chamber similar to the one introduced for direct current (DC) stimulation in Mobini et al. (2016, 2017). Experimental and numerical results are compared and an approach to cope with the electrochemical EEI is presented. A focus is laid on the relation between stimulation methods and their simultaneous potential to serve electrochemical characterisation. Techniques to feed data, which can be recorded *in situ*, into the theoretical models are introduced. We use uncertainty quantification techniques together with high-accuracy simulation methods to compute relevant observables and their expected uncertainty due to limited knowledge and/or statistical noise. We show that the resulting model has predictive power and can be used to detect undesired changes in the cell culture. Here, we demonstrate the detection of a significant temperature change introduced after the insertion of stimulation electrodes, which were kept at room temperature prior to stimulation. Eventually, we discuss how our results can be transferred into a more detailed *in vitro* or even *in vivo* model. For that, we formulate clear recommendations for experimental approaches and for reporting guidelines to ensure that realistic numerical models could be built on the basis of experimental data in the future.

## 2 Materials and Methods

### 2.1 Stimulation Chamber

The stimulation chamber has initially been described in Mobini et al. (2016, 2017). Detailed instructions to replicate the chamber have been published in Leppik et al. (2019). The chamber consists of a standard 6-well culture plate (Greiner Bio-One, Frickenhausen, Germany) with a modified lid. In each well, two platinum wires bent into L-shapes are connected to the lid and placed 25 mm apart. The upper part of the platinum wire is 18 mm long while the bottom part is 22 mm long. The wire has a cylindrical shape with a radius of 0.5 mm.

The electrodes are connected through insulation-displacement connectors and a planar multi-wire cable to a circuit board. The electrode pairs can be connected in series, in parallel, or in any other manner depending on the concrete circuit board used for connecting the electrodes to the power supply. This also permits only one electrode (pair) to be connected at a time. The circuit board is placed outside of the incubator, where the stimulation chamber is usually placed for *in vitro* experiments. Photos and technical drawings of the chamber can be found in Supplementary Figure S1.

**FIGURE 1:**
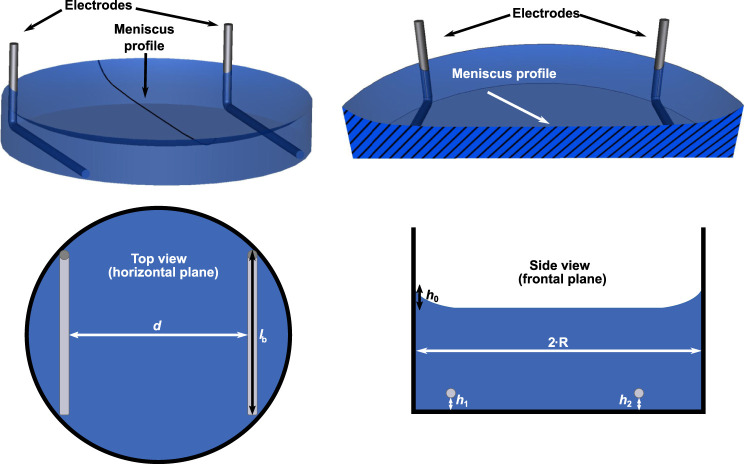
A 3D model of one well with two electrodes is shown **(upper row).** The radial profile of the meniscus was constructed using Eq. 1. The meniscus profile is highlighted in a cross-section through the centre of the well. Possible parameter choices for the uncertainty quantification (UQ) studies are indicated in sketches of the top and side view of the chamber **(lower row):** spacing between electrodes *d*, length of bottom part of electrode *l*_b_, height of electrode with respect to bottom of well (*h*_1_, *h*_2_) and height of the meniscus *h*_0_. They account for horizontal or vertical movement of the electrode or variation of the meniscus height in Eq. 1. For more details on the choice of the modelling parameters see also Table 1.

We prepared a parametrised 3D geometry of an electrode pair in a well using the open-source CAD tool *SALOME* ^1^ (Figure 1). The model generally resembles the one that we have published in Budde et al. (2019) but differs in one aspect: In this work, we also consider the meniscus profile of the cell culture medium arising because of the capillary action in the Petri dish to get closer to the experimental reality (Figure 1). The height of the meniscus has been found to follow (Schuderer and Kuster, 2003)

$$h\left(r\right)=h_{0}\left(e^{-\frac{R-r}{c}}+e^{-\frac{R+r}{c}}\right)\;,$$ (1)

where *r* is the radial distance from the centre of the well, *R* is the radius of the well, *h*_0_ is the maximum height of the meniscus relative to the height at *r* = 0 mm, and *c* is a parameter describing the decay of the meniscus. In preliminary measurements, we observed a maximum height of about 2 mm, which is about 0.4 to 0.5 mm less than previously reported (Schuderer and Kuster, 2003). However, the dish used in this work has a slightly larger radius than the dishes used in Schuderer and Kuster (2003). We did not measure the parameter *c* and assumed its value to be 2 mm as determined by Schuderer and Kuster (2003). We will address the uncertainties due to these choices later in the uncertainty quantification (UQ) approach. The height of the cell culture medium at *r* = 0 mm was determined using a bisection algorithm, which ensured a correct volume of the cell culture medium with an error of less than 0.1 µL.

**FIGURE 2:**
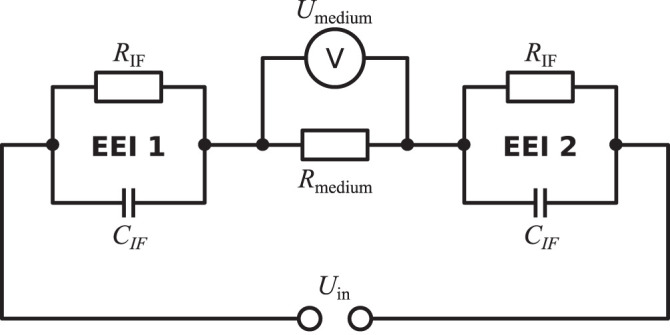
Simplified equivalent circuit to describe the electrical stimulation chamber. The electrical stimulation is delivered by the applied voltage *U*_in_. The system’s response is characterised by the impedances of the electrode-tissue interfaces (EEI 1 and EEI 2) and the impedance of the cell culture medium, which can be approximated as a resistor *R*_medium_. In this simplified scheme, the interface resistance *R*_IF_ describes charge transfer due to faradaic reactions and the interface capacitance *C*_IF_, which can alternatively be modelled by a constant-phase element representing an imperfect capacitor, describes the electrolytic double layer (Richardot and McAdams, 2002). However, there exist alternative descriptions for the EEI impedance. The voltage drop across the medium (*U*_medium_) is imposed as a Dirichlet boundary condition in the FEM simulations because the EEI impedance is *a priori* not exactly known in practice.

The electrical stimulation chamber can be approximately described by an equivalent circuit (Figure 2). In this scheme, the impedances of the wires and the electrodes themselves are omitted as their magnitude is expected to be negligibly small. The dominant contributions to the system’s impedance, which characterises the system’s response to the applied stimulation, stem from the EEIs and the resistance of the cell culture medium. The EEI impedances comprise a resistive (usually related to faradaic reactions) and a capacitive part (due to charging of the double layer). It is important to stress that the EEI impedance becomes nonlinear with increasing applied voltage *U*_in_ (Moussavi et al., 1994; Richardot and McAdams, 2002). This means that the observed impedance depends on the applied voltage in the nonlinear case. At a single frequency, the current then contains higher harmonics and possibly also a DC component (Orazem and Tribollet, 2017). Usually, there is no prior knowledge of the EEI available because it heavily depends on the electrode surface and geometry (Boehler et al., 2020).

A numerical model for the digital twin of the chamber should account for the electrochemical processes at the EEI. An example for the integration of the EEI into a finite element method (FEM) model by appropriate boundary/interface conditions can be found in Cantrell et al. (2008). Usually, the applied voltage is set on the electrode surfaces (Dirichlet boundary conditions). The voltage drop across the cell culture medium, which influences the field strength, can then be computed only if the impedances of the EEIs are known. The approach presented in Cantrell et al. (2008) relies on the assumption that the experimental results of a platinum electrode in saline at room temperature (Richardot and McAdams, 2002) can be used for any platinum electrode. This assumption is in general not valid (Boehler et al., 2020). The influence of the EEI for weak electrochemical reactions is restricted to the close vicinity of the electrode, where a diffusion layer without charge neutrality builds up. In the bulk volume, charge neutrality is preserved. The layer at the electrode surface in which the concentrations of the charged species differ from their bulk value is usually in the order of µm and has also a different pH value than the bulk solution (Auinger et al., 2011). In many cases, a change in the pH value due to the electrical stimulation would be indicated by a colour change of the cell culture medium, which can be easily detected. We make the assumption that the layer around the electrode in which the charge neutrality is not preserved is small in comparison to the dimension of the cell culture well and can be neglected in the modelling approach. Then, we can solve a numerically more efficient linear model, which we will introduce in the following, instead of an involved non-linear multiphysics model that explicitly models the interaction between applied electrical stimulation and ion dynamics (Farooqi et al., 2019).

### 2.2 Numerical Methods

#### 2.2.1 Finite Element Method

The electromagnetic fields used in the electrical stimulation of biological samples are usually considered to be slowly varying (van Rienen et al., 2005). Thus, the magnetic field is deemed negligibly small and the electroquasistatic field equation is solved

$$\nabla \cdot \left[\left(\sigma \left(\omega ,r\right)+j\omega \varepsilon \left(\omega ,r\right)\right)\nabla \Phi \left(\omega ,r\right)\right]=0\;,$$ (2)

where *σ* is the conductivity, *ɛ* is the permittivity and Φ is the electric potential, which is a phasor. In general, all quantities depend on the angular frequency *ω* and on the respective material. However, for the system considered here, this equation can be simplified. Because the surrounding air and well plate are far less conductive than the cell culture medium, they can be accounted for by an insulating boundary condition. The electrodes are not modelled explicitly but a fixed potential is assigned to each of them as a Dirichlet boundary condition, which establishes a non-zero potential drop across the cell culture medium. Furthermore, the conductivity and permittivity of the cell culture medium can be assumed as frequency-independent up to very high frequencies far above 1 MHz (see for example (Peyman et al., 2007) where the dielectric properties of NaCl, which behaves similar as a cell culture medium (Mazzoleni et al., 1986), have been investigated). In this work also aqueous KCl solution was used, for which the same holds true (Chen et al., 2003). Given that *σ* ≫ *ωɛ* for the materials and frequency range considered here (up to 5 MHz), it suffices to solve Laplace’s equation instead of Eq. 2

$$\nabla^{2}\Phi =0\;.$$ (3)

Note that in this case, the potential is a real-valued quantity (i.e., a phasor with a phase of zero). The FEM is a suitable method to solve Laplace’s equation on realistic geometries (Rylander et al., 2013). We used *NGSolve 6.2.2102* (Schöberl, 2014), which is an open-source library for higher-order FEM built on top of the mesh generator *NETGEN* (Schöberl, 1997). If not stated otherwise, we used second-order Lagrange elements and a second-order geometry representation.

This work aims at the validation of the numerical simulations to ensure that a digital twin has been found. A non-invasive technique for validation is preferable because it can potentially also be used *in situ*. An observable that can be measured non-invasively is the current through the chamber. For a potential difference *U*, the current can be computed from the power dissipation *P* (Rylander et al., 2013)

$$P=UI=\int_{\Omega }\sigma |\nabla \Phi {|}^{2}d\Omega \;,$$ (4)

where Φ is the FEM solution for the electric potential. This permits to compute the resistance of the cell culture medium, which is defined as *R* = *U*/*I*. The current can in principle also be computed by integrating the normal component of the current density over the electrode surface. In accordance with Rylander et al. (2013), we found in preliminary numerical experiments that the current computed using the surface integration converges slower. Thus, we only report results based on Eq. 4. A quantity of interest is the electric field **E**, which is defined as the negative gradient of the potential Φ (i.e., is a vector field). In previous work, we found that the field is almost homogeneous in the centre of the well, where the cells are located (Budde et al., 2019), (i.e., has only one non-zero field component). Hence, we only evaluate and report the field strength (the magnitude of the field vector), which in this particular case is equal to the non-zero component of the field at the centre of the well.

For all numerical experiments, we set the voltage difference *U* to 1 V and the conductivity *σ* to 1 S m^−1^. Thus, we will compute a reference value for the current (and resistance), which can be easily adjusted by proper scaling to match the respective experimental reality. This approach is valid because Eqs 2, 3 are linear partial differential equations. We will discuss this approach in greater detail in the Results section.

It is important to establish an estimate for the error of the numerical simulation to meaningfully interpret the results of the UQ study. Hence, we performed adaptive mesh refinement using a Zienkiewicz-Zhu error estimator Zienkiewicz and Zhu (1987) for the base geometry described in Section 2.1. From a numerical point of view, the aforementioned base geometry can be considered as the worst case. The reason for this is that this configuration features the smallest possible distance of the electrode to the dish. Thus, small elements are needed to discretise the geometry around the electrode. These elements contain a comparably large error and might need additional refinement. For other geometrical configurations, the distance of the electrode to the dish is larger and thus the numerical error is expected to be smaller. Different meshing hypotheses can be used and will eventually influence the mesh quality (Schöberl, 1997). The adaptive mesh refinement strategy on a mesh that was generated using a hypothesis to generate a very fine mesh leads to a change of the current and field strength of less than 0.01*%*. The deviation from benchmark results, which were obtained using the commercial FEM software COMSOL Multiphysics®V5.5, were equally small. Because we expect this numerical error to be much smaller than the possible uncertainty obtained in a UQ study, we used the above-mentioned meshing hypothesis for all computations.

#### 2.2.2 Uncertainty Quantification

To assess the accuracy and reliability of the numerical model, the uncertainties of the input parameters need to be propagated through the model. In practice, uncertain input parameters have to be identified and a probability distribution for each uncertain parameter needs to be specified. Then, the numerical model needs to be run multiple times to generate results that reflect the uncertainties of the input parameters. For this purpose, there exist two main approaches: Monte Carlo (MC) methods, which draw samples from the probability distributions and Polynomial Chaos (PC) methods, which generate a polynomial representation based on the probability distributions (a surrogate model) (Lemieux, 2009; Xiu, 2010). MC methods often require thousands of model runs to yield a reliable estimate of the model uncertainty and are thus not suitable for realistic 3D models. The PC approach with a point collocation method, which is a robust method in the context of PC UQ methods, requires usually far fewer model runs and was thus the method of choice (Tennøe et al., 2018). We used a modified version^2^ of the Python library *Uncertainpy* (Tennøe et al., 2018). The polynomial order was set to four. Statistical metrics such as the mean, variance or the Sobol indices, which express the influence of the uncertain parameters on the modelling outcome, were directly computed from the PC expansion. To estimate the 5th and 95th percentile, both 10^4^ and 10^5^ samples were drawn from the surrogate model to ensure convergence. To speed up the computations, the model runs were performed in parallel on the HAUMEA high-performance computing cluster of the University of Rostock (each computing node equipped with 2 Intel Xeon Gold 6248 CPUs with in total 40 cores and 192 GB RAM).

In Figure 1, possible error sources to be included in an UQ analysis are indicated. The assumed hypotheses for the UQ computations are summarised in Table 1. Note that we did not consider the effect of the cell culture because the focus in this work was on applications involving cells seeded in 2D culture. In 2D culture, the cells adhere to the bottom of the well in a very thin layer, which is a few micrometres thick. Such a thin layer is not expected to have any influence on the current through the chamber or the impedance, which are of interest in this work. Moreover, we considered only uniform distributions. This reflects our current knowledge of the uncertainties of the individual parameters. We would like to mention that our approach can be straightforwardly used with all probability distributions that are implemented in *Chaospy* including, for example, the normal distribution (Feinberg and Langtangen, 2015).

**TABLE 1:** Assumptions for the uncertainty quantification calculations. U stands for uniform distribution. We distinguish between geometrical and handling uncertainties. The geometrical uncertainties are due to manufacturing inaccuracies or the limited knowledge of the exact geometry. We estimated the geometrical uncertainties of the electrodes based on a measurement of the chamber used in this work. In contrast, the handling uncertainties are introduced by the experimenter.

Parameter	Distribution	Reasoning
**Geometrical uncertainties**		
Height of electrode *h*_1_ or *h*_2_/mm	U(0.01,1.5)	Misalignment of electrodes
Length of bottom part *l*_b_/mm	U(21,22.3)	Misshaping of electrodes
Spacing of electrodes *d*/mm	U(23,25)	Misalignment of electrodes
Decay of meniscus profile *c*/mm	U(1.95,2.05)	Estimate based on Schuderer and Kuster (2003)
Height of meniscus profile *h*_0_/mm	U(1.8,2.5)	Estimate based on Schuderer and Kuster (2003)
**Handling uncertainties**		
Cell culture medium *V*/ml	U(3.4,3.6)	Pipetting inaccuracies

### 2.3 Experiments

#### 2.3.1 Direct Current Stimulation – Chronoamperometry

The potentiostatic DC stimulation is the stimulation method, for which the above-mentioned chamber has been designed (Mobini et al., 2016). In electrochemistry, the monitoring of the time-dependent current at fixed voltage is known as chronoamperometry (Bard and Faulkner, 2001). We performed the DC measurements using the cell neurobasal medium (details are given in Section 2.3.4) inside an incubator at 37°C. Only one electrode pair in one well was used.

The potential was applied using a laboratory power supply (Voltcraft PS 405 Pro). A digital multimeter (Voltcraft VC 404) was used to ensure a constant voltage throughout the entire experiment. We used a digital multimeter (Voltcraft VC 850) together with a Bluetooth device (Voltcraft VC 810) to record the current. The sampling interval was 1 s. We applied the current for about 10 min, then short-circuited the two electrodes until the discharging current became zero and then reversed the polarity. The current was recorded for three voltages: 1 V, 1.25 V and 1.5 V (in this order). We used different stimulation chambers for the AC and DC experiments because DC stimulation caused surface oxidation, which could have caused reduced reproducibility of AC experiments. We will discuss this in the Results section.

During all measurements inside the incubator, the temperature was recorded with a thermometer. Furthermore, the temperature inside the cell culture medium was estimated by placing a temperature sensor (DrDaq, temperature sensor DD100, PicoLog 6, Pico Technology) in an adjacent well filled with the same amount of medium.

#### 2.3.2 Electrochemical Impedance Spectroscopy

Impedance spectra were recorded using a Gamry Reference 600+ potentiostat. The input amplitudes were set to 25 mV. In preliminary numerical experiments, we also used 50 mV and did not observe a visible difference, which indicates that the selected amplitude was chosen sufficiently small to exclude electrochemical reactions at the EEI. Unless stated otherwise, the spectra were recorded from 1 Hz to 5 MHz. The electrochemical impedance spectroscopy (EIS) measurements were carried out in a two-electrode configuration (i.e., no reference electrode was used). The EIS spectra were analysed using the open-source software ImpedanceFitter (Zimmermann and Thiele, 2021). By applying a linear Kramers-Kronig validity test (Schönleber et al., 2014), it was checked, which part of the spectrum could be successfully fitted to an equivalent circuit. Usually, only points at high frequencies greater than 1 MHz and at very low frequencies below 10 Hz had to be excluded from the analysis.

For the EIS experiments, we used both an aqueous KCl solution of known conductivity (HI7030, Hanna Instruments) and the cell culture medium for the characterisation of the chamber. The KCl solution was used at ambient conditions (25°C) and the cell culture medium as described in Section 2.3.4. The conductivity of the KCl solution at 25°C is 1.288 S m^−1^. The conductivity of the cell proliferation medium was measured with a handheld conductivity meter (LF 325-A, Wissenschaftlich Technische Werkstätten, Weilheim, Germany) and was 1.38 ± 0.05 S m^−1^ at 37°C.

To check if the results obtained with one electrode pair can be also used for six electrode pairs, we performed EIS measurements also using six filled wells with each 3.5 ml medium. When using six wells connected in series, the measured impedance is

$$Z=\overset{6}{\underset{i=1}{\sum }}Z_{i}\approx 6Z_{1}\;,$$ (5)

where *Z*_*i*_ is the impedance of a single well and the *Z*_*i*_ are expected to be similar to the previously measured impedance of a single well *Z*_1_. Likewise, the impedance of six wells connected in parallel is expected to be

$$Z={\left(\overset{6}{\underset{i=1}{\sum }}\frac{1}{Z_{i}}\right)}^{-1}\approx \frac{Z_{1}}{6}\;.$$ (6)

#### 2.3.3 Rectangular Wave Stimulation – Broadband Impedance Spectroscopy

We investigated the current and voltage response to pulses with a frequency of 130 Hz, which is commonly used in DBS (Krauss et al., 2020). The pulse width was chosen as 60 µs, 200 µs, or 600 µs. Both monophasic and biphasic pulses without an interphase gap were investigated.

The voltage signal was supplied by the ISO-STIM 01D unit (NPI electronics). The current signal was measured using a 1 Ω shunt resistor and amplified using a custom-built amplifier with a gain of 10. Both signals were recorded using an oscilloscope (RTB2004, Rohde&Schwarz). Note that because of the shunt resistor, not the entire input voltage drops across the stimulation chamber. To keep the influence of the shunt resistor negligible, we chose its resistance to be much smaller than the smallest expected impedance of the stimulation chamber in the relevant frequency range.

The voltage and current responses were Fourier transformed using the fast Fourier transform (FFT) method of the *NumPy* package (Harris et al., 2020). The impedance was estimated by dividing the Fourier-transformed voltage signal by the current signal. This technique is also known as broadband impedance spectroscopy (Sanchez et al., 2012). The applied voltages were chosen to be 1, 1.5 and 2 V such that the current amplitude was between 1 and 10 mA. In the current-controlled mode, the current amplitude was kept fixed at 6.5 mA.

#### 2.3.4 Cell Experiments

The stimulation chamber was tested with adult neural stem cells (aNSCs). aNSCs were prepared from the subventricular zone of the adult mouse brain and cultured essentially as described previously (Hermann et al., 2009; Walker and Kempermann, 2014). In brief, singularised cells were cultured as monolayer cultures on poly-l-ornithine/laminin coating in serum-free proliferation medium consisting of Neurobasal A medium (+1% glutamate, 2% B27 supplement, 1% antibiotic/antimycotic supplement (all from Thermo Fisher Scientific), 20 ng ml^−1^ epidermal growth factor (EGF), 20 ng ml^−1^ fibroblast growth factor 2 (FGF-2; both from Peprotech), 2 μg ml^−1^ Heparin (Sigma)). For stimulation experiments, cells were plated on 6-well cell culture plates at a density of 38 ,000 cells/cm^2^ in proliferation medium. After 4 days, neuro-glial differentation of aNSC was initiated by changing the medium to differentiation medium consisting of Neurobasal A (+1% glutamate, 2% B27 supplement, 1% antibiotic/antimycotic supplement (all from Thermo Fisher Scientific), 100 µm cAMP (from Sigma), and 10 ng ml^−1^ brain derived neurotrophic factor (BDNF; from Peprotech)) for 6 days.

Here, we report only the technical details of the cell culture stimulation approach including aspects on aNSC survival. The biological effects of the stimulation represent a separate set of experiments and will be reported elsewhere.

Different stimulation protocols were assessed: short-term stimulation (current-controlled stimulation for 30 min, one hour, 2 hours; voltage-controlled stimulation for 24 h) and long-term stimulation (current-controlled stimulation for 12 h per day for 4 days (in proliferation phase) or 10 days (4 days in proliferation and 6 days in differentiation phase)). In the current-controlled mode, symmetric biphasic pulses with 6.5 mA, 130 Hz and pulse width of 60 µs were used to simulate *in vivo* deep brain stimulation conditions (Krauss et al., 2020) and the wells were connected in series. In the voltage-controlled mode, the same waveforms were used but with an amplitude of 1.5 V and the wells were connected in parallel.

Cell viability was tested by visual inspection in short-term stimulation conditions and by quantitative cell counting in long-term stimulation experiments using DAPI staining of cell nuclei for reliable counting. During the cell experiments, the voltages and/or currents were monitored using a RIGOL DS1000Z oscilloscope. The recordings were controlled and the data saved to a laptop using the VISA interface and a self-written Python script^3^.

## 3 Results

### 3.1 Numerical Predictions of the Simulation model—a Prerequisite for the Digital Twin

Because the numerical FEM problem is linear, the relevant observables (i.e., electric field and current) depend linearly on the imposed voltage difference *U*. Let *I*_0_ be the current that is computed for an imposed voltage difference of *U*_0_ = 1 V and a conductivity of *σ*_0_ = 1 S m^−1^, then the expected current *I* at voltage *U* and (in general temperature-dependent) conductivity *σ*(*T*) is

$$I=\frac{\sigma \left(T\right)}{\sigma_{0}}\frac{U}{U_{0}}I_{0}\;.$$ (7)

Thus, the computed resistance of the cell culture medium is independent of *U* but depends on the temperature-dependent conductivity of the cell culture medium

$$R=\frac{U}{I}=\frac{\sigma_{0}}{\sigma \left(T\right)}\frac{U_{0}}{I_{0}}\;.$$ (8)

Because the UQ assumptions (Table 1) are all uniform distributions, it is sensible to propagate the 90*%* prediction interval. This means that we will establish a lower and an upper bound for each observable. To account for the uncertainty in the conductivity, we multiplied the 5th percentile with the lowest possible conductivity and the 95th percentile with the highest possible conductivity. To estimate the lowest and highest possible conductivity, we assumed a temperature fluctuation of ±1°C together with a change of the conductivity value of 2%/^◦^C. This estimate was based on the manufacturer information and is similar to values reported for cell culture media (Mazzoleni et al., 1986).

First, we ran the UQ analysis only with the geometrical uncertainties (six parameters in total) to assess the uncertainty arising from the manufacturing process. With six parameters, 422 runs were required (a formula to compute the number of runs for a given polynomial order and number of uncertain parameters can be found in (Tennøe et al., 2018)). These runs were usually done within a few hours thanks to a high degree of parallelism. We found that the meniscus decay had almost zero influence (Supplementary Figure S2). Thus, we omitted it from the further analysis.

**FIGURE 3:**
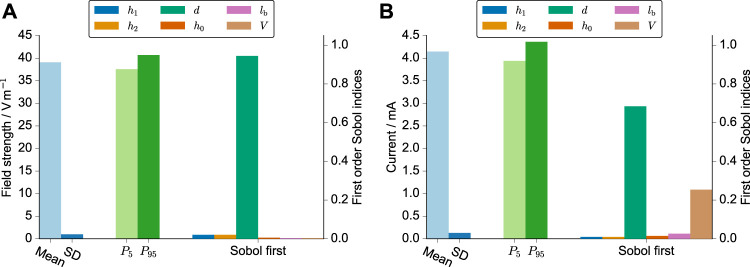
UQ results for the electric field strength **(A)** and the current **(B)**. The mean, standard deviation, 5th and 95th percentile are shown together with the first-order Sobol indices, which indicate the individual influence of the respective parameter on the simulation result. The varied parameters were the height of the meniscus profile *h*_0_, the height of the left and right electrodes *h*_1_/*h*_2_, the spacing of the electrodes *d*, the length of the horizontal part of the electrode *l*_*b*_, and the volume of the cell culture medium *V*. These parameters and their probability distributions are explained in greater detail in Table 1. The simulations were run for an imposed voltage difference of 1 V and a conductivity of 1 S m^−1^.

Then, we considered the uncertainty of the volume due to pipetting inaccuracies. Changes in the volume appeared to have an influence on the current but not on the field strength (Figure 3). The error of the spacing (about 4*%*) is almost linearly propagated through the model for the electric field strength. This is also highlighted in the probability distributions of the samples drawn from the surrogate model (Supplementary Figure S3). The distribution of the field strength appears to be almost uniform, thus indicating that the assumed probability distribution for the electrode spacing is dominantly influencing the uncertainty of the predicted field. In contrast, the distribution of the current is widened and more bell-shaped, which highlights the additional influence of the volume uncertainty. Eventually, we used only the three parameters, which influenced the current the most (*d*, *l*_*b*_, *V*), to reduce the number of required simulation runs to 72. The uncertainty estimate did then not deviate notably from the previous results, while the UQ analysis takes considerably less time.

In Table 2, we report the 90*%* prediction interval of the resistances *R* as well as the prediction intervals multiplied with the uncertainty interval of the conductivity. The prediction intervals for the field strength in V m^−1^ were [37.56, 40.67] for 3.5 ml, [36.87, 39.85] for 4 ml, and [35.58, 38.45] for 5 ml. These results suggest a slight decrease in the field strength with increasing volume.

**TABLE 2:** Comparison between predicted resistance of the medium *R*_medium_ and *R*_medium_ as extracted from fits to experimental data (more details in Section 3.2.2). The values are reported in Ω. The fitted and measured impedance deviated on the order of the accuracy of the potentiostat (1*%*) indicating the high quality of the fit. We did not investigate the experimental error in greater detail and thus estimate it to be 1*%* for all reported values. The predicted values (between the 5th and 95th percentile) are entirely based on the UQ analysis. The uncertainty of the conductivity *σ* was assumed to be ±2*%* of the expected value (1.288 S m^−1^ for KCl at 25°C, 1.38 S m^−1^ for cell culture medium at 37°C). The values for parallel and series connections were estimated using Eqs 5, 6.

Electrolytic solution	Volume	Experimental	Predicted	Predicted with uncertainty of *σ*
KCl	3.5 ml	183.84	[177.97, 197.18]	[174.48, 201.21]
KCl	4.0 ml	167.26	[157.42, 173.39]	[154.34, 176.93]
KCl	5.0 ml	138.40	[129.58, 141.96]	[127.04, 144.85]
Medium (1-well)	3.5 ml	166.56	[166.11, 184.04]	[162.85, 187.79]
Medium (6-well)	3.5 ml, ∥	29.02	[27.69, 30.67]	[27.14, 31.29]
Medium (6-well)	3.5 ml, series	1075.25	[996.66, 1104.24]	[977.10, 1126.74]

In the following, we will present experimental approaches to augment the model by an EEI impedance and assess its predictive power. In particular, we will assess if the corresponding experimental observations lie within the aforementioned prediction intervals. The experimental approaches are briefly summarised in Table 3.

**TABLE 3:** Relation between methods of electrical stimulation and electrochemical characterisation methods and their relevance for the numerical model.

Stimulation signal	Characterisation method	Relevance
DC current/voltage	Chronopotentiometry/Chronoamperometry	Monophasic signals contain DC component
Sine wave	Electrochemical Impedance Spectroscopy (EIS)	Frequency sweep permits to characterise entire system (Figure 2), needed to augment FEM model
Rectangular pulse	Broadband impedance spectroscopy	Like EIS, but simultaneous measurement at many frequencies, needed to calibrate numerical model, compare to predictions

### 3.2 Stimulation Methods for Characterising the Stimulation Chamber and Augmenting the Simulation Models—Constructing the Digital Twin

#### 3.2.1 Direct Current stimulation—Chronoamperometry

We observed that the measured current did not grow linearly with the applied voltage. This would have been the expected behaviour for a circuit dominated by the ohmic resistance of the cell culture medium. Instead, the current drastically decreased with increased stimulation time. Even after about 10 minutes, the currents did not converge to a steady value. We could describe the recorded current response *I* by a function of the following form:

$$I\left(t\right)=\frac{a}{\sqrt{t}}+be^{-t/c}\;,$$ (9)

where *a*, *b*, and *c* are positive constants and *t* is the time. The equation could describe a superposition of a faradaic, diffusion-limited current inversely proportional to $\sqrt{t}$ and nonfaradaic, capacitive current decaying with exp(−*t*) (Bard and Faulkner, 2001). The nonfaradaic current can, for example, be interpreted in terms of a charging of the double layer or the pseudocapacitance at the electrode surface (Merrill et al., 2005; Lasia, 2005). However, more advanced measurements would be required to unambiguously explain the observed behaviour. The behaviour of platinum during electrical stimulation is still subject of ongoing research (Hudak et al., 2017). Importantly, one cannot establish a direct relation between Eq. 9 and the cell culture medium resistance, which can be computed from the FEM solution. Thus, the current recorded at a fixed voltage cannot be used to validate numerical simulations based only on Eq. 3. Instead, local potential recordings would be required (Gundersen and Greenebaum, 1985).

**FIGURE 4:**
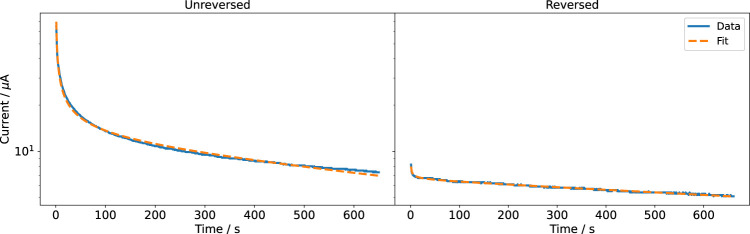
Recorded and fitted currents through the cell culture medium at a DC voltage of 1 V. Note that the ordinate is log-scaled because the current decreases sharply with time.

Eq. 9 was fitted to the experimental data using a nonlinear least-squares method. The fitted current was in good agreement with the measured current for all voltages (Figure 4, Supplementary Figure S4, S5). Studying the two parts of Eq. 9 individually (Supplementary Figure S6) revealed that before reversing the polarity, the faradaic and nonfaradaic currents are on the same order of magnitude. The nonfaradaic current is almost constant at all times. During the measurement period, the current did not converge to this constant value. After reversing the polarity, the influence of the faradaic current is considerably smaller. It even seems as if the current after polarity reversal was a continuation of the current before polarity reversal. Because we short-circuited the electrodes and thus there should be no residual charge stored in the system before reversing the currents this observation is surprising. The result suggests an electrochemical memory of the system. This could mean that both electrodes are continuously changed during the experiment and that the state of the electrodes is not reversed when changing polarity. There might also be other reasons for the irreversibility; for example, depletion of reactive species around the electrodes (Boehler et al., 2020). Then, the composition of the medium around the electrodes could have changed and a diffusion layer, which we do not include in our simulation model, could be present. When using the stimulation chamber in cell experiments, we observed a change of the colour of the anode, most likely showing oxidation (PtO_2_). Thus, we cleaned the electrodes electrochemically after each DC stimulation application by applying a higher voltage of *U* = 5 V for 5 min in NaCl. For the cell experiments, this ensured replicable stimulation currents.

#### 3.2.2 Electrochemical Impedance Spectroscopy

**FIGURE 5:**
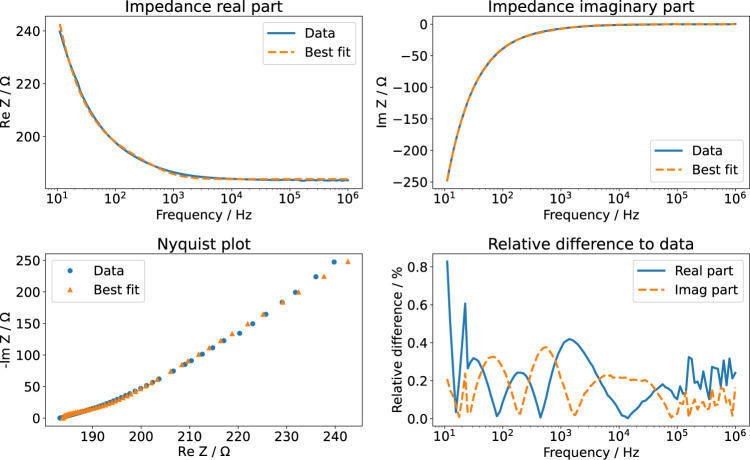
EIS measurement of aqueous KCl solution (3.5 ml). The real part and imaginary part of the measured data are compared to the best fit results using the impedance model of Ragoisha et al. (2010). The relative difference is given with respect to the absolute value of the impedance.

The equivalent circuit shown in Figure 2 distinguishes between the two EEIs. However, the EEIs are in practice indistinguishable unless a reference electrode is used. Hence, the EEIs are often described by one circuit comprising a constant-phase element (CPE) in parallel with a charge-transfer resistance (Richardot and McAdams, 2002). We found that this equivalent circuit did not describe the EIS spectra well. Instead, we used a circuit that had been developed to describe platinum surface oxidation (Supplementary Figure S7); more explanations on the involved elements are given in the Supplementary Material, Supplementary Figures S8, S9) (Ragoisha et al., 2010). Then, the fitted impedance values deviated usually less than 1*%* from the experimental data (an example is shown in Figure 5). For some configurations, an additional lead inductance improved the fit results. This was particularly the case for the 6-well configuration. The ohmic resistance *R*_medium_ can be directly compared to the numerical simulations (Table 2). All measured values lie within the prediction intervals of the numerical simulation, which validates our model. Using different volumes of the KCl solution showed that the liquid could be modelled entirely as a resistor: the measured imaginary part did not depend on the volume, which would have been expected if the imaginary part would not be exclusively due to the EEI (Supplementary Figure S10). The cutoff frequency where the impedance changes from capacitive to resistive behaviour can be estimated to lie between 1 and 10 kHz. We will later show the impact of this quantity on the current and voltage transients. In sum, these results show that the numerical simulations can reliably predict the ohmic resistance of the culture medium while the EEI properties can only be inferred from EIS measurements.

It is known that the EEI impedance behaves nonlinearly with increasing voltage amplitude at low frequencies (i.e., less than 1 kHz) (Moussavi et al., 1994; Richardot and McAdams, 2002). Thus, we checked the impedance at the fundamental frequency (130 Hz) for increasing voltage amplitudes. Indeed, we could observe nonlinear behaviour (Supplementary Figure S11). The impedance did not change notably at amplitudes lower than 250 mV. Hence, this voltage amplitude can be used as an estimate for the limit of linearity.

#### 3.2.3 Rectangular Wave Stimulation—Broadband Impedance Spectroscopy

Rectangular waves can be described in the frequency domain by Fourier series (see also Supplementary Material). This reveals that the frequencies used in therapeutic applications such as DBS also contain high frequencies (Gimsa et al., 2005). To obtain the frequency-domain representation of the signals, there exist two popular approaches: fast Fourier transform (FFT) of time-domain signals (Butson and McIntyre, 2005) or the use of the analytically available expressions for the Fourier series (Butenko et al., 2020). The main difference in the frequency spectra of the waveforms considered in this work is that the biphasic pulse has its main contribution at higher frequencies than the monophasic pulse (Supplementary Figure S12–14). The amplitudes of the individual frequency components of the different waveforms did not exceed the aforementioned limit of linearity for an overall pulse amplitude of 1 V. Amplitudes greater than or equal to 2 V would lead to frequency amplitudes greater than 250 mV and thus potentially non-linear responses at low frequencies.

We used the FFT approach to estimate EIS spectra from time-domain data. The impedance is given by

$$Z\left(\omega \right)=\frac{U\left(\omega \right)}{I\left(\omega \right)}$$ (10)

with *ω* the angular frequency, *Z* the impedance, *U* the potential and *I* the current in the frequency domain. The impedance computed by the FFT is known at equally spaced frequencies. The frequency resolution (i.e., the frequency spacing) depends on the length of the time signal. We found that recording about 10 periods, which corresponds to a frequency resolution of about 10 Hz, yielded a sufficient resolution. We used a truncation method to reduce noise in the FFT spectra: only data points with a current amplitude that is at least 10*%* of the maximum current amplitude were considered. This approach has also been proven to be effective for numerical simulations (Butenko et al., 2019).

To construct time-domain signals under consideration of the measured EIS spectra, we used the analytical Fourier series approach. Note that charge-balancing signals such as symmetric biphasic, biphasic with delay etc. can be straightforwardly computed as the superposition of time-shifted monophasic rectangular waves.

In preliminary experiments, we found that the current-controlled monophasic waveform with KCl showed a problem of a large DC voltage offset (Supplementary Figure S16). Moreover, the EIS spectra changed after using such a waveform, which indicated a possible change of the electrodes as also mentioned in Section 3.2.1 (data not shown). Hence, we did not further consider current-controlled monophasic waveforms because potentially harmful electrochemical reactions cannot be excluded. Simple reasoning for this DC offset, which has also been reported elsewhere (Paap et al., 2021), can be given based on the results presented in Section 3.2.1. The employed monophasic pulse with an amplitude of 6.5 mA, a pulse width of 60 µs, and a frequency of 130 Hz has a DC component of 50.7 µA. We found that a DC voltage of about 1 V caused a current of only about 10 µA decaying with time (Figure 4). This explains why the current-controlled monophasic waveform required a voltage DC offset greater than 1 V.

**FIGURE 6:**
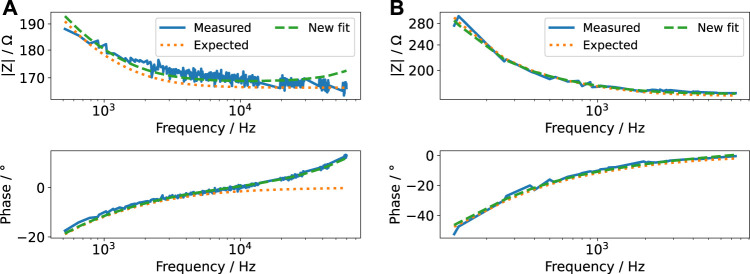
Bode plot of the impedance computed from FFT of voltage and current pulse for a pulse width of 60 µs **(A)** and 600 µs **(B)** and an amplitude of 2 V (i.e., for voltage-controlled mode, the currents are shown in Figure 7 and for current-controlled mode, the voltages are shown in Figure 8) (*measured*). Note that we omitted Fourier components with small magnitude to reduce noise at higher frequencies. The measured impedance is compared to the impedance expected after EIS measurements (*expected*). A new fit to the measured impedance was made by varying only the lead inductance, ohmic resistance of the cell culture medium and double-layer capacitance (*new fit*). Note that both abscissa and ordinate differ between the figures because the signals comprise different frequency contributions. The ordinate is log-scaled.

The impedance data obtained using the FFT algorithm (Figure 6) could be well explained using the EIS results from Section 3.2.2. Nevertheless, the impedance deviated slightly from the impedance measured by EIS. Thus, we fitted the impedance again to update the parameter values of the impedance model (Supplementary Figure S7). Considering a change in all variables turned out to be an inappropriate approach. Some waveforms contain information only in a limited frequency range (Supplementary Figure S12-14). In this case, not all parameters of the impedance model could be unambiguously determined. Some fit parameters were linearly correlated. In consequence, this caused a wrong estimate of the ohmic resistance. Instead, we found that permitting changes in 1) inductance, 2) ohmic resistance and 3) double-layer capacitance sufficed to accurately describe the measured data. The increased lead inductance (evidenced by the positive phase of the measured data in Figure 6) is most likely caused by the long and unshielded wires connecting the stimulator and the stimulation chamber. Because the conductivity of the medium depends on the temperature, the deviation of the expected and observed ohmic resistance can be, for example, explained by the uncertainty of the incubator’s temperature control. The change in the double-layer capacitance was usually only a few per cent. It could be caused by an electrochemical reaction at the electrode surface (Ragoisha et al., 2010), which occurs due to the applied electrical stimulation. With this result, we established a means to update the model (Supplementary Figure S7) based on evolving data, as required for a digital twin (Wright and Davidson, 2020). Furthermore, it permits to identify (undesired) changes in the stimulation system with increasing stimulation time.

**FIGURE 7:**
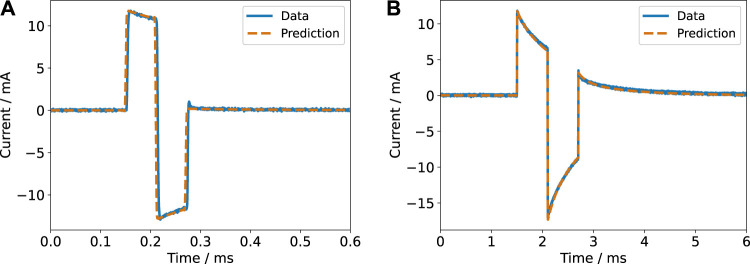
Current response for a biphasic pulse in voltage-controlled regime (2 V amplitude) with a pulse width of 60 µs **(A)** and 600 µs **(B)** for a single electrode pair. The experimental data is compared to the prediction based on the impedance model.

**FIGURE 8:**
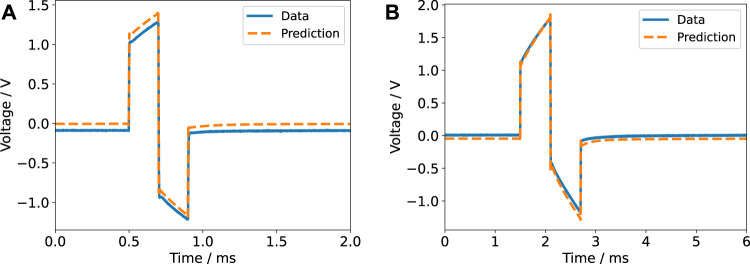
Voltage response for a biphasic pulse in current-controlled regime (6.5 mA amplitude) with a pulse width of 200 µs **(A)** and 600 µs **(B)** for a single electrode pair. The experimental data is compared to the prediction based on the impedance model. A DC offset of unknown origin is evident in the left panel and is also present in the right panel. Most likely, the offset was caused by the stimulator.

For the (re-)construction of the stimulation signals, two modes have to be considered: voltage- and current-controlled stimulation. Either the voltage or the current pulse is controlled to be a rectangular pulse. Having the parameter values to compute the impedance at hand, we estimated both signals in the frequency domain using their Fourier series representation and Eq. 10. We observed a very good agreement between theory and experiment (Figures 7, 8). Particularly, the voltage and current transients in voltage-controlled mode could be predicted by the fitted parameter values of the impedance model (Figure 7 and Supplementary Figure S17). The measured currents show the influence of the cutoff frequency, which is deemed to be one important characteristic of a stimulation electrode (Boehler et al., 2020). The signals with dominant contributions at frequencies greater than the cutoff frequency (Figure 7A and Supplementary Figure S18A) yield a more rectangular current than the signals with a longer pulse width and thus more dominant low-frequency components.

Notable deviations between the prediction and the recorded data were only observed in the current-controlled mode and when the six wells were connected in parallel. The voltage in the current-controlled biphasic stimulation set-up revealed a DC offset (Figure 8). The offset could be reduced by manually tuning the stimulator before each experiment but could still amount to about 100 mV due to limited tuning accuracy. The biphasic signal does not comprise a DC component and thus we suppose that the DC offset stems from a coupling capacitor in the stimulator (van Dongen and Serdijn, 2016). Other authors have also reported a similar observation, which was termed DC contamination (Neudorfer et al., 2021). Due to the small magnitude of the DC offset, it does not significantly affect the current through the medium and is thus not identifiable without monitoring the voltage. Furthermore, without comparison to the digital twin prediction, it could possibly be overlooked. Nevertheless, the offset might still cause continuous electrochemical reactions.

While the current-controlled monophasic pulses revealed a very large DC voltage offset (Supplementary Figure S16), we observed a negative DC current offset for voltage-controlled monophasic pulses. This was most evident when using KCl solution (Supplementary Figure S17) instead of the medium (Supplementary Figure S18). In contrast to the current-controlled mode, the DC current offset was in good agreement with the theoretical prediction (Supplementary Figure S17, 18). The impedance predicted by the model (Supplementary Figure S7) tends to infinity when the frequency tends to zero (i.e., to the DC limit). Then, the (positive) DC current component is blocked by an infinitely large DC impedance (see Eq. 10). Because the DC current component does not contribute to the current signal, a significant (negative) DC offset can be observed. In terms of electrochemistry, the DC offset indicates an infinitely large charge transfer resistance, which suggests that no significant faradaic electrochemical reactions occur (Richardot and McAdams, 2002). Due to the blocking effect, the cells would be exposed to a small field in the negative direction even when no signal is actively applied (i.e., when the input voltage is zero). For this reason, current-controlled pulses are often preferred over voltage-controlled pulses because the applied stimulation field strength is proportional to the current density but not to the applied voltage. The aforementioned DC voltage offset that appears for current-controlled monophasic pulses can then be removed using charge-balancing approaches (Paap et al., 2021), which we did not cover in this work.

In the case of the parallel connection, it turned out that the waveform slightly deviated from the expected waveform (Supplementary Figure S19). The impedance of the 6-well system connected in parallel is only about 30 Ω. Hence, the total current through the system becomes large (about 60 mA) and might negatively affect the performance of the stimulator (Tandon et al., 2009). This result highlights the importance of a digital twin for the performance assurance of the electrical stimulation device. Still, the agreement between prediction and the measured current was good (Supplementary Figure S19B). By integrating the shunt resistor (1 Ω) into the equivalent circuit model (Supplementary Figure S7) and repeating the analysis, we could study its influence. At this point, the shunt resistor did not significantly change the results, but we will discuss later a case, where the shunt resistor has to be modelled explicitly.

When connecting the six wells in series, we did not observe similar behaviour as for the parallel connection (data not shown). This indicates that the observed deviations are indeed explained by the small load impedance of the parallel connection. In general, the good agreement between predicted and measured voltage and current transients for the parallel and series connection is highly important because it suggests that there is no significant difference between the individual electrode pairs with respect to their electrochemical behaviour.

Validating the FEM simulations enabled us to establish a connection between macroscopic quantities (voltage, current) and local quantities (potential, field strength). This permits estimating the field strengths to which the cells are exposed from the current transients. For that, the voltage drop across the medium *U* (i.e., the boundary condition of the simulation) is computed by multiplying the measured current *I* and the computed resistance *R* (known from Eq. 8 for a known conductivity *σ*). We use the UQ bounds for the resistance *R* (Table 2) to obtain error bounds for the voltage drop *U*. For each *U*, the prediction interval for the field strength is known (see Section 3.1). Because this approach requires knowledge of the current *I* and the conductivity *σ*, it is termed current-conductivity method.

There is a second way to estimate the field strength inside the cell culture medium through the estimation of the voltage drop across the cell culture medium *U*_medium_. This approach requires exact knowledge of the impedance of the medium *Z*_medium_ to apply the voltage divider formula

$$U_{\text{medium}}\left(\omega \right)=\frac{Z_{\text{medium}}\left(\omega \right)}{Z\left(\omega \right)}U_{\text{in}}\left(\omega \right)\;.$$ (11)

**FIGURE 9:**
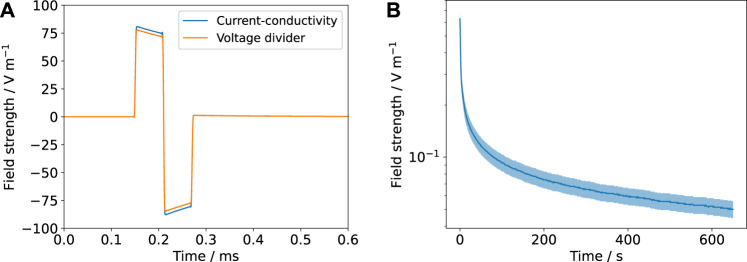
The estimated field strengths using the current-conductivity method (based on Eq. 8 and Table 2) and the voltage-divider approach based on an equivalent circuit scheme (Figure 2 and Supplementary Figure S7) are compared. The mean value is shown (solid line) with the prediction interval (shaded). **(A)** corresponds to Figure 7A and **(B)** to Figure 4. Note that for the DC result **(B)**, there is no possibility to estimate the field through the voltage-divider approach because no suitable equivalent circuit model is available.

The total impedance *Z* is known from the fitted EIS spectra and/or fits to the Fourier-transformed voltage/current transients. This approach, which we refer to as the voltage-divider approach, has one advantage over the previously presented current-conductivity approach: the error of the conductivity does not need to be considered and thus the field estimates are more accurate (Figure 9).

The same field estimate procedure can be applied for wells connected in series or parallel. However, then the error estimates are less reliable because the computation is done using the approximation that all electrode pairs share the same impedance (see Eqs 5, 6). We attempted to account for this by a worst-case assumption: the lower bound for the impedance is computed under the assumption that all wells have the minimal impedance computed for one well. Vice versa, the maximum impedance of one well was used to estimate the upper bound for the impedance. For the voltage-divider approach, we did not include an additional error estimate and used the error bounds for the field strength determined for one well. This approach probably underestimates the error.

**FIGURE 10:**
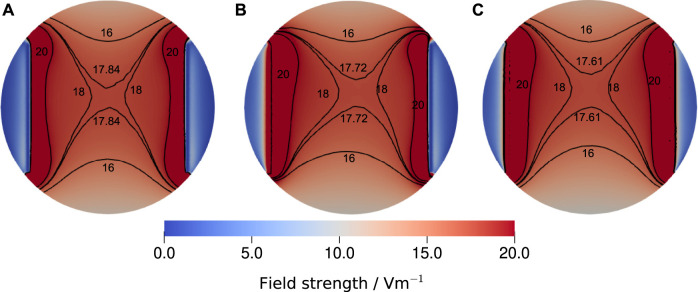
Comparison of the electric field strength at the bottom of the well for the largest experimentally determined EEI impedance at 130 Hz, which was 195.33 Ω, a medium conductivity of 1.38 S m^−1^, a volume of 3.5 ml and a stimulation voltage of 1 V. Three different configurations were considered: **(A)** the voltage-divider approach, where the voltage drop across the medium has been computed for the given impedance, **(B)** the asymmetric distributed configuration, where the Robin boundary condition (Eq. S9) was applied only on the left electrode using the full impedance and **(C)** the symmetric distributed configuration, where the EEI impedance was divided by two and applied on both electrodes. The reference voltage Φ_ref_ was equal to the voltages chosen for the Dirichlet boundary conditions. For the sake of comparability, the isolines for 16 V m^−1^, 18 V m^−1^ and 20 V m^−1^ are shown together with the isoline for the field strength at the centre of the well that we reported throughout this manuscript. Evidently, the three modelling approaches yield only slightly different results. Thus, we concluded that the lumped approach, which permits to estimate the field strength without repeat simulations, delivers a sufficiently good estimate of the field strength. More information on the simulation approach are given in the Supplementary Material.

The presented approach for estimating the electric field strength based on an equivalent circuit model is usually referred to as lumped-element approach. Other options are distributed impedance models (Cantrell et al., 2008; Howell et al., 2014), which integrate the EEI impedance as a Robin boundary condition into the FEM model (more details are given in the Supplementary Material). Thus, they require simulation runs for each EEI impedance. Furthermore, they suffer from the problem that the EEI impedance of the individual electrodes is not unambiguously known. For the chamber studied here and the EEI impedances for the considered frequency range, we found no significant difference between the lumped and distributed approach (Figure 10), which suggests that the field estimates by the lumped-element approach are reliable. Figure 10 shows also the homogeneity of the electric field.

### 3.3 Observations During the Stimulation of Adult Neural Stem Cells—Digital Twin at Work

We tested the predictions of our model against data recorded during *in vitro* stimulation of aNSCs. We found very good agreement of theory and experiment for both voltage- and current-controlled stimulation over a period of 12 and 24 h, respectively (Supplementary Animations S1, S2; further details are given in the descriptions of the animations). These results indicate that the stimulation system is electrochemically stable over the course of the stimulation and that the stimulation does not induce a temperature increase. For the voltage-controlled stimulation, six wells were connected in parallel. To increase the load impedance, a 100 Ω shunt resistor was added, which stabilised the signal. Because the shunt impedance is greater than the impedance of the wells, it needed to be explicitly included in the model. The good agreement of theory and experiment shows that this can be done straightforwardly without harming the predictive power of the model. Naturally, the shunt resistor has to be considered when estimating the field strengths using the voltage-divider approach. A pleasant effect of a larger shunt resistor is the increased voltage drop, which makes it possible to record the current without an amplifier.

Visual inspection of cell cultures after short-term stimulation as reported in the Methods section revealed no differences in cell counts and morphology between stimulated and non-stimulated cultures. Consistently, analyses of cell numbers during aNSC proliferation phase in the centre of the stimulation well after 4 days (130 Hz, 60 µs, current-controlled at 6.5 mA, 12 h per day) showed no morphological changes of the cells and similar cell survival in stimulated (3,530 ± 460 cells/mm^2^) versus non-stimulated cultures (3,621 ± 590 cells/mm^2^; *p* = 0.923, unpaired two-sided *t*-test; *n* = 5). Similar results were obtained after 10 days of stimulation during proliferation and differentiation of aNSCs with no significant differences of cell counts in stimulated (2,835 ± 554 cells/mm^2^) versus non-stimulated cultures (3,113 ± 587 cells/mm^2^; *p* = 0.760, unpaired two-sided *t*-test; *n* = 6).

During the short-term experiments, we made an unexpected observation: when the system was not thermally equilibrated (i.e., the electrodes were kept at room temperature prior to the stimulation and were inserted into freshly changed medium just before the stimulation), the measured signals deviated from the predicted signals (Supplementary Figure S20 and Supplementary Animations S3). The peak-to-peak voltage decreased about 15% from approximately 7.5–6.5 V over a time course of about 2 h (Supplementary Figure S20). Because the signal with a pulse width of 60 µs is dominated by the ohmic resistance of the cell culture medium, we can assume that the resistance of each well also changed by about 15%. Using the aforementioned change of the conductivity of about 2*%*/°C, we can estimate under the assumption of a spatially homogeneous temperature distribution that the temperature in the well was initially decreased by approximately 7.5°C. Estimating the mixture temperature (see Supplementary Material) does not support the hypothesis that the temperature drop could have been caused by the electrodes alone, which were kept at room temperature. Instead, it is likely that the temperature of the pre-heated cell culture medium was below 37°C. Additionally, the ambient temperature in the incubator dropped during the handling. Even though the measured relaxation time seems to be surprisingly large, it appears to be credible. We observed in the validation experiments that it takes about 30 min to re-equilibrate the temperature of the cell culture medium after handling it outside the incubator (data not shown). For example, when the stimulation chamber was handled at room temperature for a few minutes, the temperature of the medium decreased from 37°C to about 33°C.

Of course, the temperature estimate needs to be refined because we only considered the average but not the local temperature, which could be inhomogeneous. Our results show that the thermal equilibration of fresh cell culture medium takes a considerable amount of time. In contrast, heating of the medium due to the applied electrical stimulation can be ruled out and does not need to be modelled because the stimulation voltage did not change within 24 h of stimulation of a thermally equilibrated system (Supplementary Figure S20). A change in temperature leads to altered stimulation conditions. For example, at lower temperatures, an increased voltage is required to drive the preset current, and this increased voltage, in turn, temporarily causes a higher stimulation field strength. In addition, a decreased temperature has an impact on the activity of excitable cells (Loppini et al., 2021). Again, this result highlights the possibilities of performance assurance using a digital twin while suggesting its extension in the direction of multiphysics modelling for future research. To sum up, we prepared a provenance graph showing all experimental and modelling steps needed for a digital twin of the stimulation device (Supplementary Figure S21).

## 4 Discussion

Electrical stimulation has been (re-)discovered as a tool for tissue engineering and regenerative medicine (Balint et al., 2013; da Silva et al., 2020). In recent years, the effect of electrical stimulation on various cell lines and tissues has been studied *in vitro* (Funk et al., 2009; Balint et al., 2013; Jahr et al., 2015; Thrivikraman et al., 2018; Chen et al., 2019; da Silva et al., 2020; Ryan et al., 2021). Nevertheless, no clear picture regarding optimal stimulation parameters (field strength, stimulation voltage/current) has been developed. Recently, the variety of proposed DC stimulation protocols has been scrutinised and it has been suspected that many reported field strengths values may have been overestimated (Guette-Marquet et al., 2021). This problem has also been identified for magnetic field stimulation (Portelli et al., 2018). In this work, we suggest building a digital twin of an electrical stimulation system. The digital twin comprises an *in silico* model of the stimulation chamber, which is calibrated by prior electrochemical characterisation, and can be updated dynamically through analysis of the stimulation waveforms, which can be recorded *in situ*. Eventually, this approach aims at enabling performance assurance and reproducible research. Particularly the *in silico* modelling extends the guideline for stimulation experiments suggested by Boehler et al. (2020).

Having established a validated model and defined a clear relation between the important observables in time as well as in frequency domain, we are able to formulate our main insights and limitations regarding the effectively delivered electrical stimulation:

1) The naive estimate of the field strength is: *E* = *U*/*d*. In this approach, the geometry of the well and the electrodes is mostly neglected and the field is assumed to be spatially homogeneous, which is a valid estimate only for parallel-plate capacitor geometries with sufficiently large electrodes. Moreover, it is often assumed that the voltage drop across the medium *U* is equal to the voltage delivered by the stimulator. For the chamber considered here, the estimated field strength ranges between 40 V m^−1^ and 43.5 V m^−1^ for 1 V (and thus 80 V m^−1^ to 87 V m^−1^ for an amplitude of 2 V). Because this approach does not require information on the impedance of the system and/or on the voltage and current transients, it may lead to insufficient documentation of the stimulation experiment.
2) By acquiring more information on the geometry, studying the dielectric properties of the system and monitoring both the voltage and current transients, a validated and comprehensible simulation model can be built. The validated simulation yields a spatially-dependent field strength. For the part of the well where the cells are located, the field strength can be estimated to range between about 65 V m^−1^ and 90 V m^−1^ for a rectangular pulse of 2 V amplitude, a frequency of 130 Hz and a pulse width of 60 µs when using a medium volume of 3.5 ml (based on the current-conductivity method). Due to the EEI impedance, the time course of the field strength depends strongly on the frequency and pulse width. For the DC stimulation at 1.5 V, the current (and thus the field) decays rapidly over time and the asymptotic field strength can be approximated as about 0.05 V m^−1^. With the voltage-divider method, the field cannot be estimated for the DC stimulation because no impedance model is available. For the stimulation using rectangular pulses, the voltage-divider method is applicable and yields a field ranging between about 70 V m^−1^ and 80 V m^−1^, which is a more accurate estimate than obtained by the current-conductivity method. These results can be straightforwardly updated if a different medium volume is used or the frequency and/or pulse width are changed.
3) Still, the model leaves room for improvement. Local recordings of the voltage would be ideal to corroborate our results. For that, microelectrode arrays could be integrated into the well. For systems, where, unlike in this work, the simulation results for the electric field indicate a significant difference between the lumped and distributed modelling approach, such local measurements are inevitable. Local pH measurements would be important to identify electrochemical processes (Pfau et al., 2018). We assumed the influence of possible ion movements, cell layers and cell volume fraction to be negligible. Local field/impedance measurements would be required to refine the models regarding effects on the cellular scale (for example, by employing optical methods (Pucihar et al., 2006; Yang et al., 2021)). Also, local temperature fluctuations were not yet studied by us.

We could show that a linear model describes the experimental data well when rectangular pulses are used. Thus, we can assume that no strong electrochemical reactions occur in this case. The quantities needed for the model (EEI impedance, conductivity) could be determined accurately prior to the actual stimulation experiment and can, in principle, be monitored and updated *in situ*. The model can be used to predict voltage and current transients, which are, for example, relevant to estimate neural activation (Holsheimer et al., 2000). Moreover, properties such as the charge per phase, which can be used to design safe stimulation protocols (McCreery et al., 1990; Merrill et al., 2005), can be estimated prior to the stimulation experiment. Hence, the number of experiments could possibly be reduced by identifying unsafe stimulation parameters at an early stage.

At the current stage, we could not find a predictive model for DC stimulation. Thus, the choice of field strengths for future DC experiments cannot be supported by our model. Our experimental results for DC stimulation indicate that different electrochemical processes can be expected to occur and that the observed current is dominated by processes at the electrode surface but not by the bulk volume (which is actually relevant to estimate the effect of the stimulation). To build a meaningful model of DC stimulation we expect to require at least a non-linear formulation, which depends on the overpotential (Cantrell et al., 2008) and describes the secondary current distribution. Probably even models considering the individual ion concentrations and their temporal dynamics could be required (Farooqi et al., 2019). For models also considering secondary current densities stemming from non-linear faradaic electrochemical reactions, kinetic reaction parameters need to be known. For the chamber discussed here, such an approach has been presented in Srirussamee et al. (2021) to model DC stimulation. The model considering secondary current densities has relied on empirical data and could not predict measurement results. We measured time-dependent currents for DC stimulation, which has not been considered in the model presented in Srirussamee et al. (2021). In contrast, we can explain the measurements with rectangular pulses based on a well-understood model that comprises both identifiable contributions from the EEI and the bulk volume. Hence, we suppose that the spatial distribution of the potential and field is accurately predicted by the simulations.

The validated model can be extended by models that estimate the effect of electrical stimulation. Examples are the computation of transmembrane potential changes upon electrical stimulation (Pucihar et al., 2006) or the electromechanical interaction through either deformation of the cell (Shamoon et al., 2018) or induced motion of membrane constituents such as lipid rafts (Lin et al., 2017) or cytoskeleton proteins (Hart et al., 2013). For general *in vitro* tissue engineering experiments, network models have been devised (Geris et al., 2018). Electrical stimulation could be integrated as a factor into such models by, for example, using the stimulation field strength as a model parameter. First ideas to relate the stimulation field strength and cell differentiation and proliferation have been presented in (Dawson et al., 2020).

Even though we demonstrated the approach only for a relatively simple model system, it is relevant for laboratory practice. Advantages of the approach are that it is 1) easy to implement, 2) relies exclusively on free and open-source software, 3) uses affordable hardware. The hardware could be shipped as a small and portable solution and the data evaluation can be performed in an automated manner. Regarding the hardware, the current measurement could be improved by using a better amplifier. Potentially, the approach can be integrated into implantable stimulators such as presented in Paap et al. (2021). In comparison to the guideline suggested in Boehler et al. (2020), we did not determine the water window of the electrode system (i.e., the potential region in which neither water oxidation nor reduction occurs). However, we would like to stress that stimulation outside the water window could not be described by a linear impedance model due to the electrochemical reactions (Richardot and McAdams, 2002) and would thus be easily detected in our approach. In that, our approach is similar to the pulse-clamp method (Hung et al., 2007). Furthermore, it can probably be fed by information from time-domain electrochemistry analysis, which has been proposed as an *in situ* sensor for neural implants (Weltin et al., 2020). In comparison to the approach that has been recently suggested by Abasi *et al.* (Abasi et al., 2020), our approach does not necessarily require an impedance analyser as the monitoring unit but could be realised with only a shunt resistor connected to an amplifier and an oscilloscope. A monitoring impedance analyser scans a broad frequency range with low-amplitude sine waves, which is slower than the broadband impedance spectroscopy approach, might consider more frequencies than necessary and does not cover possible nonlinear stimulation effects (unless it is programmed to match the amplitudes of the stimulation pulse). Thus, the impedance analyser can monitor the electrochemical state of the electrodes (and cell culture) before and after stimulation but does not necessarily contribute to an understanding of the electrochemistry due to the stimulation pulse. Still, it is a good possibility to initially calibrate the numerical model and to cross-validate the results of the broadband impedance spectroscopy data. It should be integrated if possible and affordable because it offers also a better resolution than the broadband impedance spectroscopy approach (compare, for example, Figure 5, 6). Rectangular pulses, despite their proven effectiveness for electrical stimulation, are not the optimal choice for broadband impedance spectroscopy (Creason et al., 1973; Sanchez et al., 2012). In future research, optimised pulses could be used, for example, once a day to monitor the state of the stimulated sample.

Thanks to the digital twin, possible variations of the voltage/current transients can be related to different processes. As we demonstrated, temperature changes of the cell culture medium could be detected. Even though the temperature of a cell culture should be ideally kept constant, this aspect has not been mentioned in previous works using the chamber considered here (Mobini et al., 2016, 2017; Srirussamee et al., 2019). To use the ohmic resistance as a temperature sensor, the temperature dependence of the conductivity of the cell culture medium has to be known well. To date, only limited data are available (Mazzoleni et al., 1986). A database with high-accuracy data for different cell culture media should be established. Furthermore, changes in the ohmic resistance could serve as an indicator for medium contamination (growth of bacteria) or a change in the chemical constitution (ion concentrations). On the other hand, the digital twin of the stimulation chamber could be used to infer the (unknown) conductivity of cell culture media via Eq. 7. However, the resolution of the inferred conductivity is currently limited by the geometrical uncertainties. The results of the UQ analysis indicate possible improvements of the experimental set-up. The chamber considered here should be improved with respect to the accuracy of the electrode spacing. When preparing the experiments, attention should be paid to the volume of the medium in each well to ensure well-interpretable current measurements.

Changes in the double-layer capacitance could indicate electrochemical reactions at the electrode surface (Ragoisha et al., 2010). We would like to note that the term “double-layer capacitance” might be misleading as the equivalent circuit model might also describe an adsorption capacitance (Lasia, 2005), whose contribution cannot be distinguished from ionic contributions without further investigation. It has been argued that elevated primary current densities at higher frequencies could benefit corrosion (Cantrell et al., 2008). The use of biphasic pulses prevents corrosion because the electrochemical reactions are reversed. Moreover, we would expect to observe a significant change of the EEI impedance if the surface corrodes (Orazem and Tribollet, 2017). Thus, our approach might also serve as an early indicator for an electrode replacement. Furthermore, we would expect to not be able to describe the signals anymore by the linear impedance model upon corrosion (Bosch et al., 2001). We are not aware of any research relating the (non-)linearity of the EEI impedance to biologically relevant quantities such as the pH value. This will be subject of future research. DC stimulation has been shown to increase the hydrogen peroxide level in the cell culture medium (Srirussamee et al., 2021). A raised hydrogen peroxide concentration benefits corrosion (Boehler et al., 2020). Thus, monitoring and reporting of both stimulation voltage and current is imperative to ensure the reproducibility of DC stimulation studies. In our lab, we found better reproducibility of AC stimulation in comparison to DC stimulation because of the aforementioned oxidation of the electrodes in the DC regime.

For a general electrode system, a comparison between the lumped-element approach and the distributed-impedance approach is necessary to quantify the effect of the EEI impedance on the electric field. A change in the EEI impedance would then necessitate new simulations. Hence, it is recommended to choose stimulation signals with dominant contributions at frequencies greater than the cutoff frequency, from which the EEI impedance has almost no effect. In this work, this applies to biphasic pulses with a pulse width of 60 µs. Vice versa, the stimulation electrode should be chosen such that it has a low cutoff frequency (as already argued by Boehler et al. (2020)) to gain flexibility with regard to the stimulation signals.

**FIGURE 11:**
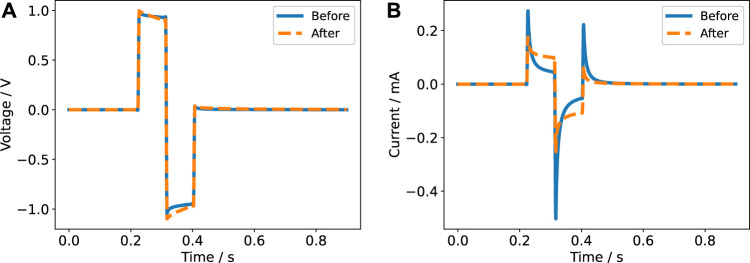
Voltage **(A)** and current transients **(B)** estimated from the *in vivo* impedance reported in Lempka et al. (2009) as measured before and after DBS application for 60 min. The voltage is the voltage drop across the tissue and the current is the current through the monkey brain. Note that we chose a symmetric biphasic stimulation pulse as an example. However, in the original publication Lempka et al. (2009) it was only mentioned that a charge-balanced biphasic waveform has been used (without further details), which could also be an asymmetric biphasic waveform.

The presented approach has relevance also if the sample contains more than one phase (e.g., hydrogel or tissue in cell culture medium). Measurements of rectangular voltage and current pulses (i.e., similar to stimulation pulses) have been used as an *in situ* method to infer the impedance of the porcine brain *post mortem* (Poßner et al., 2020). Lempka *et al.* have used EIS (i.e., sine waves unrelated to stimulation pulses) to measure the impedance of DBS electrodes implanted in rhesus macaque monkey brain *in vivo* before and after application of DBS (Lempka et al., 2009). They have interpreted the impedance in terms of a tissue and an electrode-tissue interface (ETI) component and reported a corresponding equivalent circuit model. Hence, we can perform the same analysis and predict the current transient and the voltage drop across the tissue for a symmetric biphasic DBS pulse (Figure 11). Interestingly, the voltage drops almost completely across the tissue component and no significant losses are caused by the ETI component. This indicates that the stimulation signal comprised mainly frequency components above the cut-off frequency, which was characteristic of their electrode. The predicted signals deviate significantly before and after the application of DBS. It is an interesting perspective for future research to investigate if DBS stimulation pulses measured in clinical practice can be predicted by the suggested linear model. This could pave the way for electrochemical *in situ* monitoring based on easily available information. In principle, the parameters of, for example, the impedance model suggested in Lempka et al. (2009) could then be inferred from the stimulation pulses as described by us. The consequently accessible voltage drop across the tissue could serve as an input for realistic anatomical simulation models of the brain to estimate the electric field distribution and neural activation (Lempka et al., 2018; Butenko et al., 2020; Krauss et al., 2020). Potentially, such electrochemical monitoring could also improve closed-loop stimulation approaches, which aim at adapting the stimulation parameters to react to changes in the system and yield an optimal stimulation outcome. Currently, most of the markers for closed-loop stimulation are biologically motivated (Fleming et al., 2020) and could be complemented by electrochemical information.

Furthermore, data-driven *in silico* models relevant for tissue engineering (Lesage et al., 2018) could be coupled to or extended by the circuit model presented here. In the context of data-driven models, inverse methods could be employed to estimate, for example, the dielectric properties of the cell culture medium (or hydrogels or tissue samples). In this work, we presented the forward approach: based on prior knowledge, we built a digital twin of the stimulation chamber and validated it by experimental data under consideration of the model uncertainties. However, this approach is not feasible anymore if there is no suitable prior knowledge available. This could be, for example, the case for hydrogels with dielectric properties changing over time (Mawad et al., 2016) or biological tissues with highly uncertain dielectric properties (Zimmermann and van Rienen, 2021). Then, the recorded voltage and current could be used to infer these dielectric properties. Nevertheless, the validation and verification approach presented here would still be required to establish the relationship between experiment and theory.

The methods used in this study could be straightforwardly integrated into community standards to contribute to improved documentation of experiments. Community standards are required for frameworks to improve reporting standards, which have been identified as crucial for reproducibility (Glasziou et al., 2014; Macleod et al., 2021). The chamber considered here stands as an example for improvable reporting standards in the field of electrical stimulation for tissue engineering. It has been promised to deliver a stimulation of 100 V m^−1^ at a stimulation amplitude of 2.2 V (DC) (Mobini et al., 2016). In subsequent experimental studies, this field strength has been reported (sometimes without mentioning the applied voltage) (Mobini et al., 2017; Srirussamee et al., 2019) and has thus made it into literature reviews (Thrivikraman et al., 2018; Ryan et al., 2021). However, the theoretical model presented in Srirussamee et al. (2021) has predicted a current density of 0.5 A/m^2^. This value corresponds to a field strength of only 0.33 V m^−1^ for the reported conductivity of 1.5 S m^−1^. Because the theoretical model presented in Srirussamee et al. (2021) has been in good agreement with experimental data, this field strength appears to be credible. The field strength we found in the DC setting at a lower voltage is even smaller, thus supporting the results of Srirussamee et al. (2021). Only when using rectangular pulses, field strengths approaching the reported 100 V m^−1^ could be reached. In sum, our results are in line with the conclusion of Guette-Marquet et al. (2021) that many field strengths in the literature appear to be overestimated. In future experiments, theoretical analysis of the electrochemical systems, which electrical stimulation devices inevitably are, together with thorough *in situ* monitoring appear to be paramount. Otherwise, the results of *in vitro* studies will not be comparable and will not advance the status quo.

## Data Availability

The datasets presented in this study can be found in online repositories. The name of the repository and accession number can be found below: Zenodo (dx.doi.org/10.5281/zenodo.5189259).

## Footnotes

1
https://www.salome-platform.org/

2
https://github.com/j-zimmermann/uncertainpy

3
https://github.com/j-zimmermann/PyVISAScope

## Supplementary Material

Figures S1–S21 are enclosed in Supplementary Material.

Animation S1 (VIDEO 1.FLV) Animation of the measured and predicted stimulation current in voltage-controlled stimulation using six wells connected in parallel filled with each 3.5 mL in series with a shunt resistor of 100 Ω. The duration of the stimulation was 24 hours. The timestamp of each frame is given in minutes and shown in each frame title. The predicted signal was generated using 1000 harmonics.

Animation S2 (VIDEO 2.FLV) Animation of the measured and predicted stimulation voltage in current-controlled stimulation using three wells connected in series filled with each 4 mL. Prior to stimulation, the electrodes were kept at incubator temperature (37°C). The stimulation was applied for 12 hours. Note that we used resistances derived from the UQ results (Table 2) because we did not perform EIS for this set-up. The timestamp of each frame is given in minutes and shown in each frame title. The predicted signal was generated using 1000 harmonics.

Animation S3 (VIDEO 3.FLV) Animation of the measured and predicted stimulation voltage in current-controlled stimulation using three wells connected in series filled with each 4 mL. Prior to stimulation, the electrodes were kept at room temperature. The stimulation was applied for 30 minutes, 90 minutes and 120 minutes (in total four hours). Again, the resistance estimated from Table 2 was used. The timestamp of each frame is given in minutes and shown in each frame title. The predicted signal was generated using 1000 harmonics.
