# Supplementary material for: Using a Digital Twin of an Electrical Stimulation Device to Monitor and Control the Electrical Stimulation of Cells in vitro
Source: Front Bioeng Biotechnol. 2021 Dec 8;9:765516. doi: 10.3389/fbioe.2021.765516 (PMC8693021; doi:10.3389/fbioe.2021.765516)
Supplement: Supplementary file 2 [file DataSheet1.pdf]

Supplementary Material

1 STIMULATION CHAMBER

A

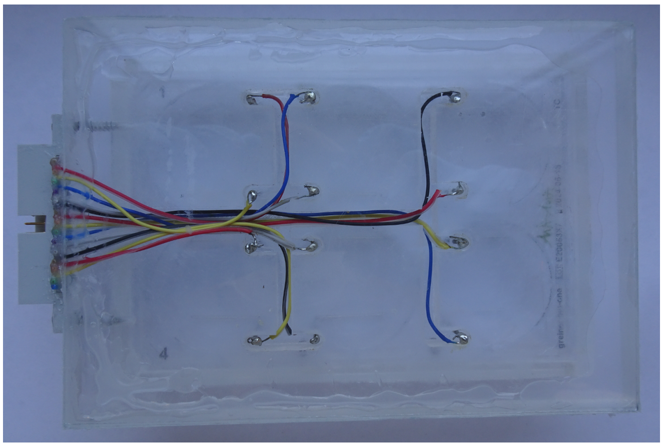

B

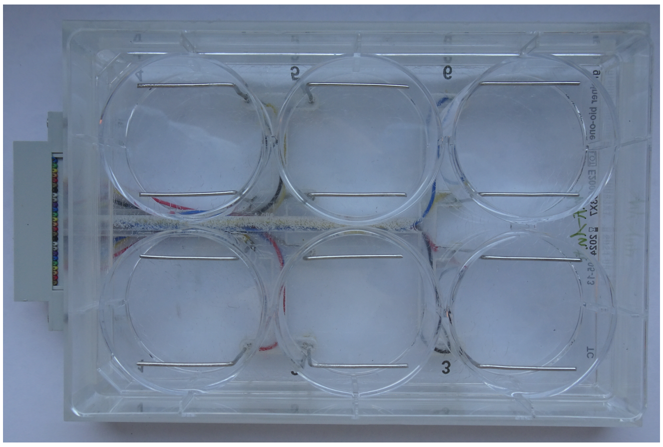

C

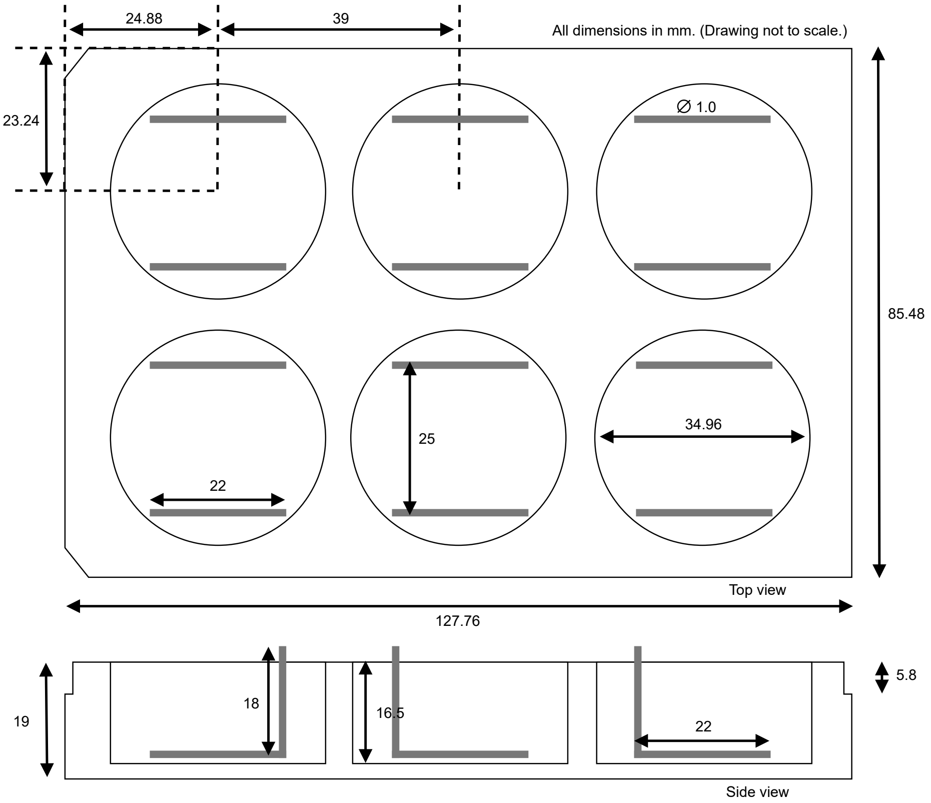

Figure S1: Images of the stimulation chamber (6-well lid with platinum electrodes) designed for flexible stimulation applications (top view **A** and bottom view **B**) as well as a drawing of the device (**C**).

2 UNCERTAINTY QUANTIFICATION

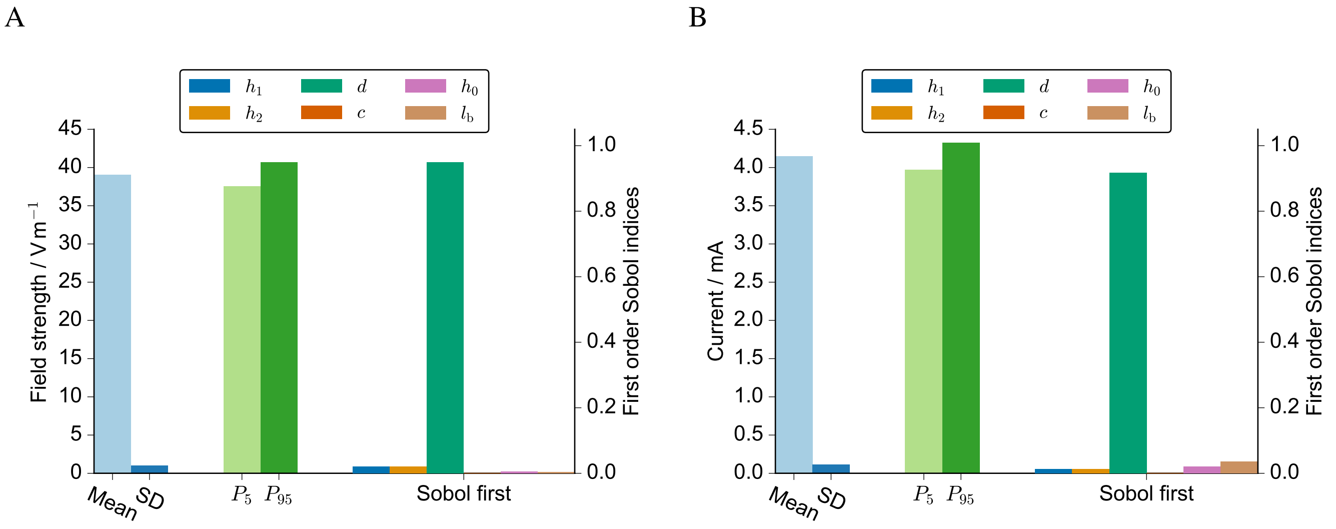

Figure S2: Comparison of UQ results for field (**A**) and current (**B**) using only geometrical uncertainties. This analysis led to an exclusion of the parameter c from further analysis.

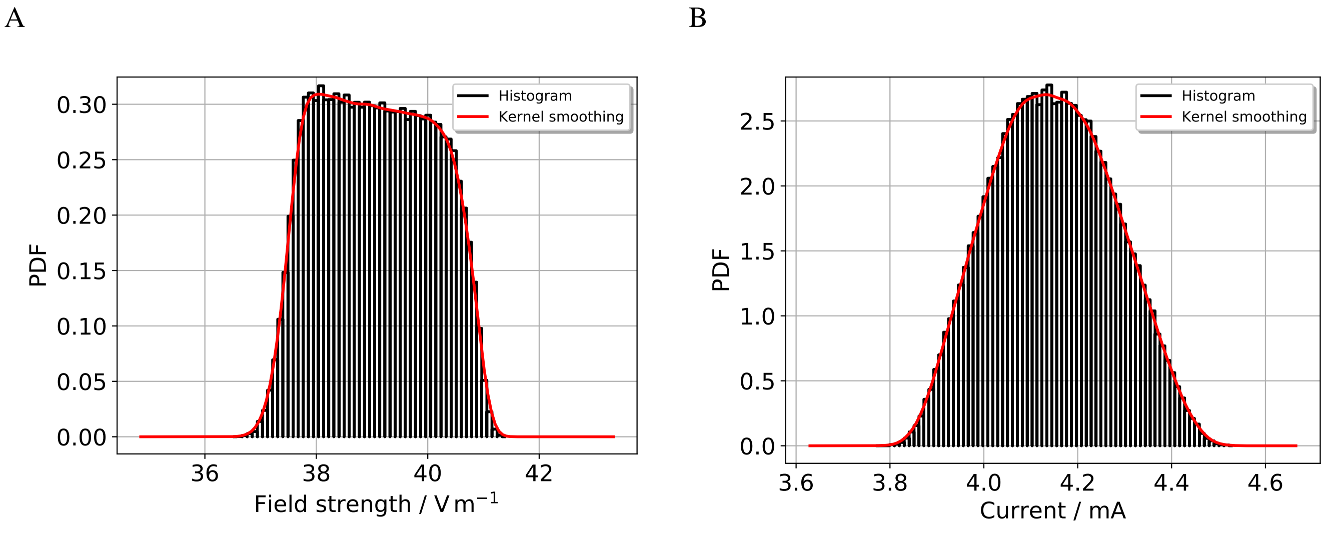

Figure S3: Comparison of probability distributions sampled from surrogate UQ models for field (**A**) and current (**B**). Note that this figure corresponds to the UQ results presented in Figure 3 in the manuscript.

3 DIRECT CURRENT STIMULATION – CHRONOAMPEROMETRY

We could describe the recorded current response I by a function of the following form:

$$I(t) = \frac{a}{\sqrt{t}} + be^{-t/c}, \tag{S1}$$

where a, b, and c are positive constants and t is the time. The equation could describe a superposition of a faradaic, diffusion-limited current inversely proportional to $\sqrt{t}$ and nonfaradaic, capacitive current decaying with $\exp(-t)$ (Bard and Faulkner, 2001).

The numerical results for the fit parameters are summarised in Table S1.

Setting	a / µA$\sqrt{\text{s}}$	b / µA	c / s
1.0 V	61.50 ± 0.29	8.22 ± 0.06	1088.84 ± 19.25
1.0 V, reversed	1.52 ± 0.03	6.45 ± 0.01	2656.98 ± 15.15
1.25 V	124.47 ± 1.53	10.42 ± 0.30	$1\,501\,934.43 \pm 167\,992\,859.52$
1.25 V, reversed	7.41 ± 0.04	13.91 ± 0.01	3095.69 ± 12.71
1.5 V	105.95 ± 0.72	21.21 ± 0.14	4181.92 ± 252.44
1.5 V, reversed	6.98 ± 0.06	21.20 ± 0.01	4624.93 ± 24.69

Table S1. The parameters obtained by fitting the experimental data to Equation (S1). Note that the large value for c, which also has a large uncertainty, for an applied voltage of 1.25 V indicates that this parameters could not be reliably estimated. This could, for example, suggest that the non-faradaic current in this particular case is time-independent because the term $\exp(-t/c)$ of Equation (S1) tends to one for $c \to \infty$.

The results for the currents recorded at voltages other than 1 V are shown in Figures S4 and S5.

The faradaic and nonfaradaic contributions to the current are shown separately for each used voltage in Figure S6.

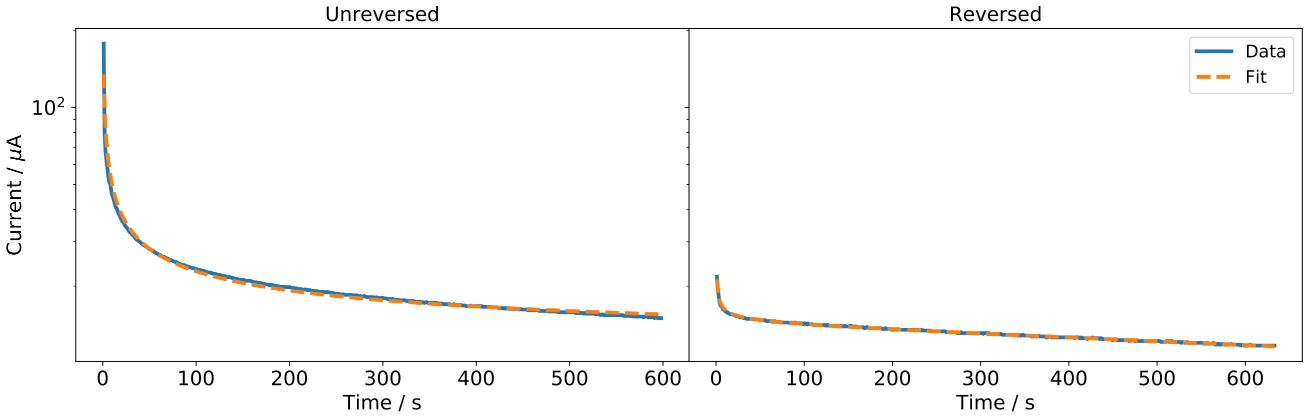

Figure S4: Recorded currents at a fixed voltage of 1.25 V together with results of fit to Equation (S1). The current with reversed polarity is shown in the right panel for comparison.

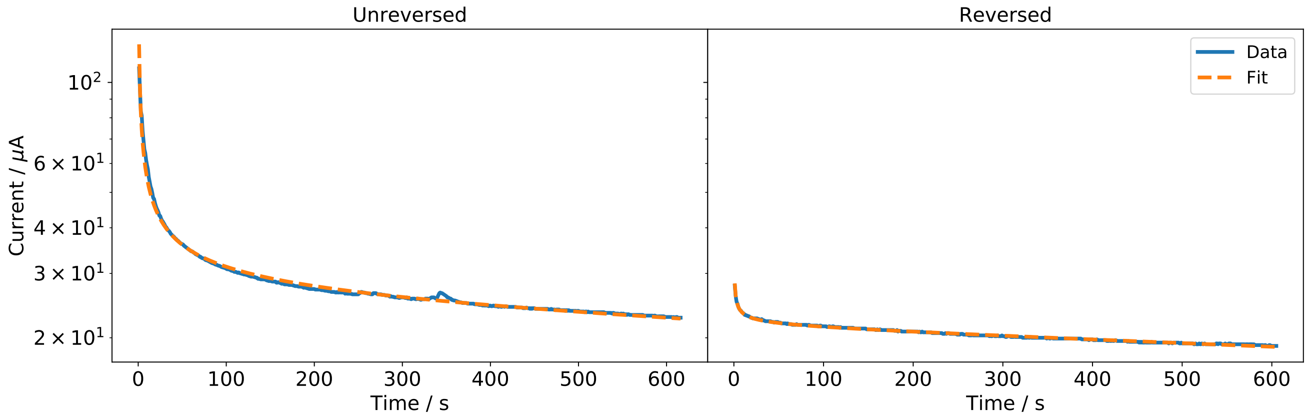

Figure S5: Recorded currents at a fixed voltage of $1.5\,\mathrm{V}$ together with results of fit to Equation (S1). The current with reversed polarity is shown in the right panel for comparison.

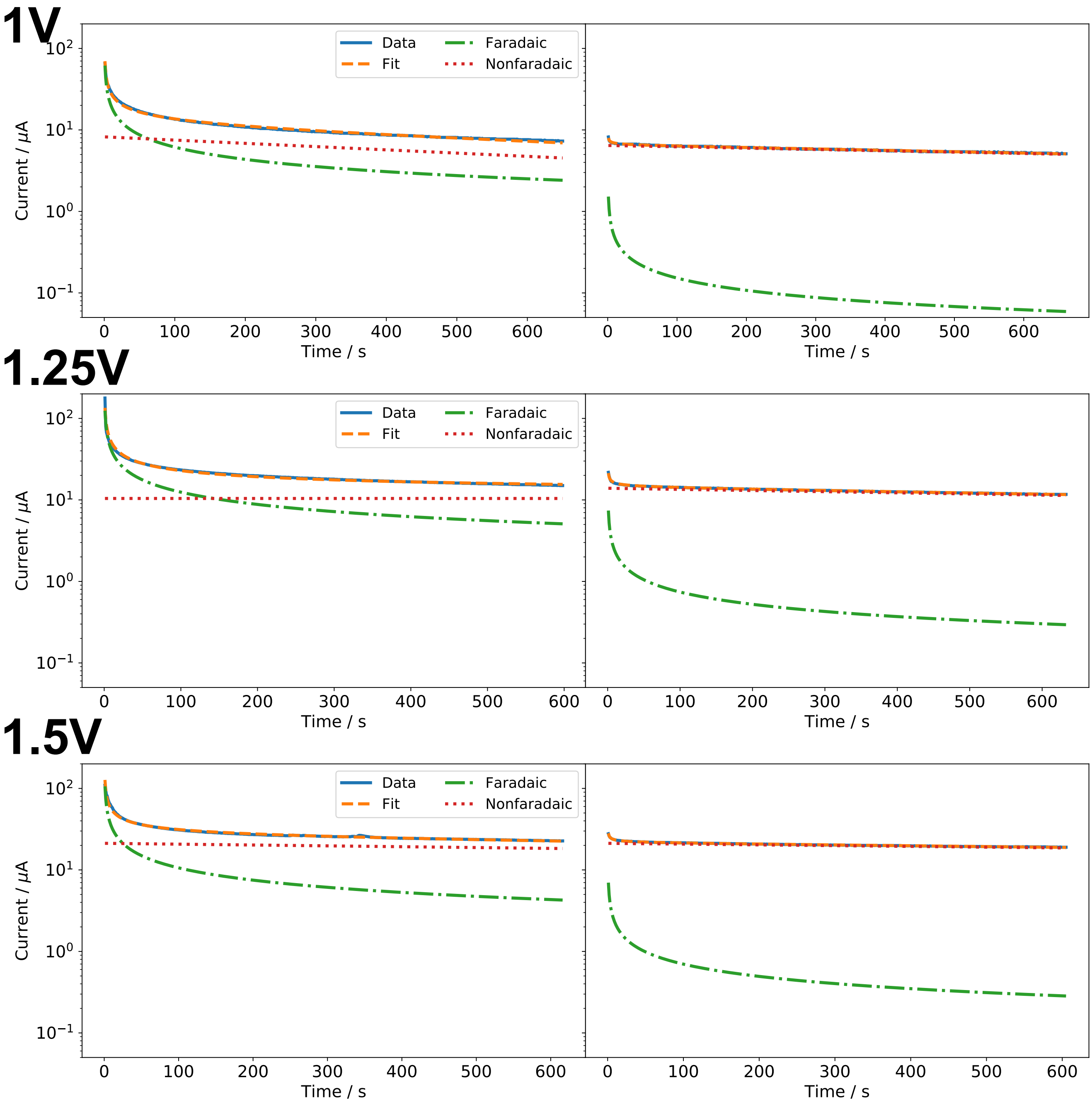

Figure S6: Recorded currents at fixed voltages of 1 V, 1.25 V and 1.5 V together with results of fit to Equation (S1) and the individual contributions of the fitting function. The current with reversed polarity is shown in the right panel for comparison.

4 FITTING OF IMPEDANCE SPECTRA

We used an equivalent circuit model described in Ragoisha et al. (2010) and shown in Figure S7. If applicable, we used a lead inductance in the equivalent circuit model. The lead inductance was required when we did not directly connect the potentiostat to the chamber. The basic elements of the circuit (Figure S7) and their characteristic frequency response are shown and explained in Figures S8 and S9. Evidently, the shape of the impedance in the Nyquist plot in Figure 5 in the manuscript cannot be described by a linear superposition of a resistor and a CPE because it cannot be described by a straight line with a fixed slope. Similarly, the impedance computed from the FFT response (shown in Figure 6 of the manuscript) has to contain contributions from an inductance because its phase turns positive at high frequencies.

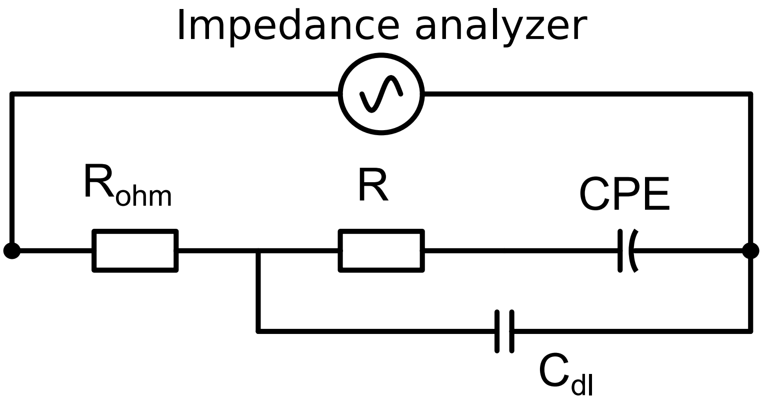

Figure S7: Equivalent circuit model as described in Ragoisha et al. (2010). One part of the interface impedance is modelled by a constant-phase element (CPE) in series with a resistor (R). The other part is modelled by a capacitor (C_{dl}), which is meant to account for the ionic double layer. The ohmic resistance of the electrolyte (R_{ohm}) is connected in series with the interface element. To highlight the source of the ohmic resistance, we also refer to it as R_{medium}. If applicable, a lead inductance can be connected in series with the ohmic resistance.

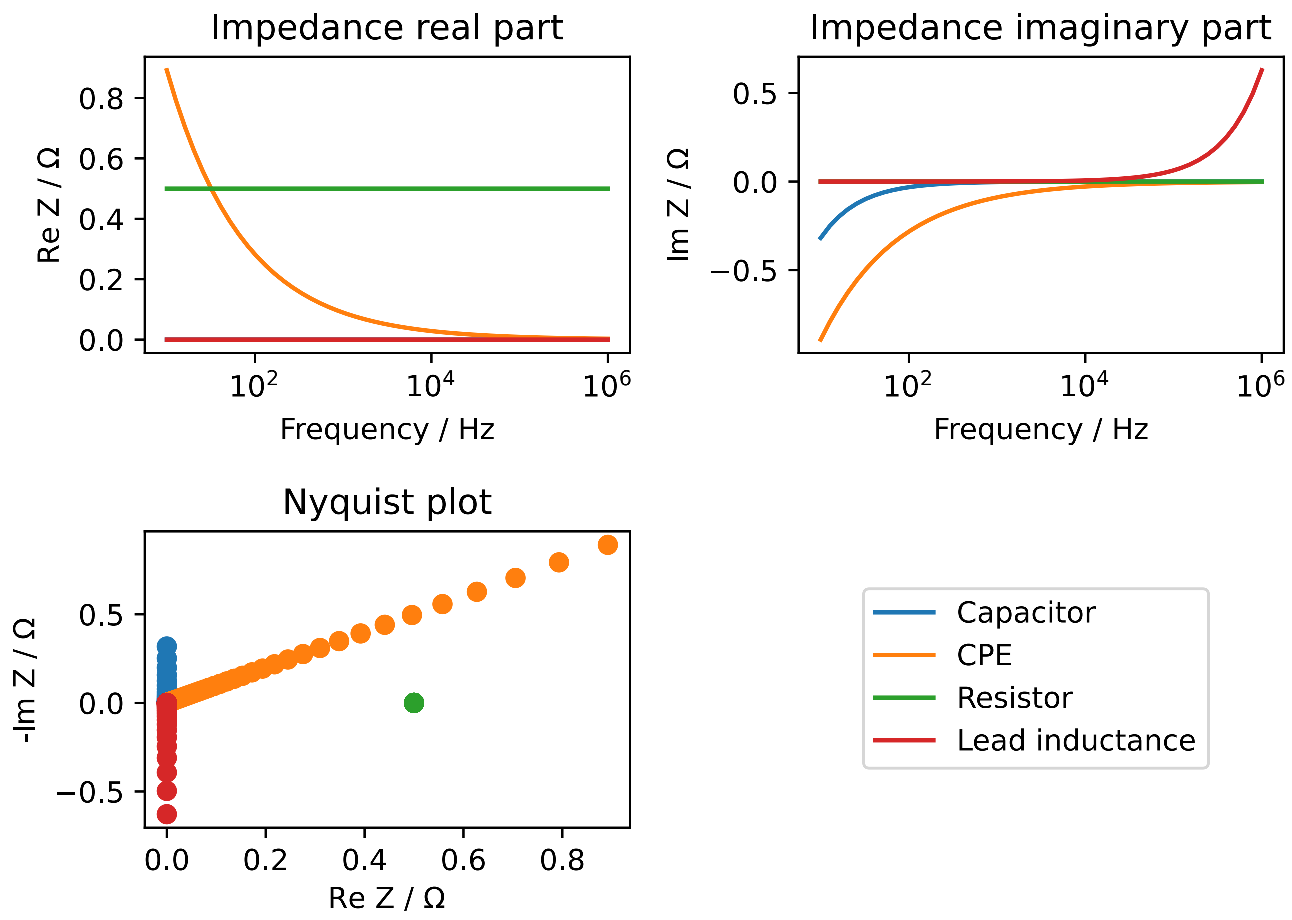

Figure S8: Impedance response of the basic elements of the circuit shown in Figure S7 for a capacitor of $50\,\mathrm{mF}$, a CPE (described by $\kappa(j\omega)^{-\alpha}$) with $\kappa = 10$ and $\alpha = 0.5$, a resistor of $0.5\,\Omega$ and a lead inductance of $100\,\mathrm{nH}$. The values were chosen arbitrarily. This figure is solely intended to highlight the impedance response of the basic elements in the relevant frequency range. The capacitor has a complex-valued impedance with zero real part and negative imaginary part, which appears as vertical line in the Nyquist plot. The CPE has a complex-valued impedance with both non-zero real and imaginary part, which appears as a straight line with a fixed slope starting from the origin in the Nyquist plot. The resistor has a real-valued impedance, which appears as a single point in the Nyquist plot. The inductance has a complex-valued impedance with zero real part and positive imaginary part, which appears as vertical line in the Nyquist plot. The impedance response of a real system is given by linear and parallel combinations of these basic elements.

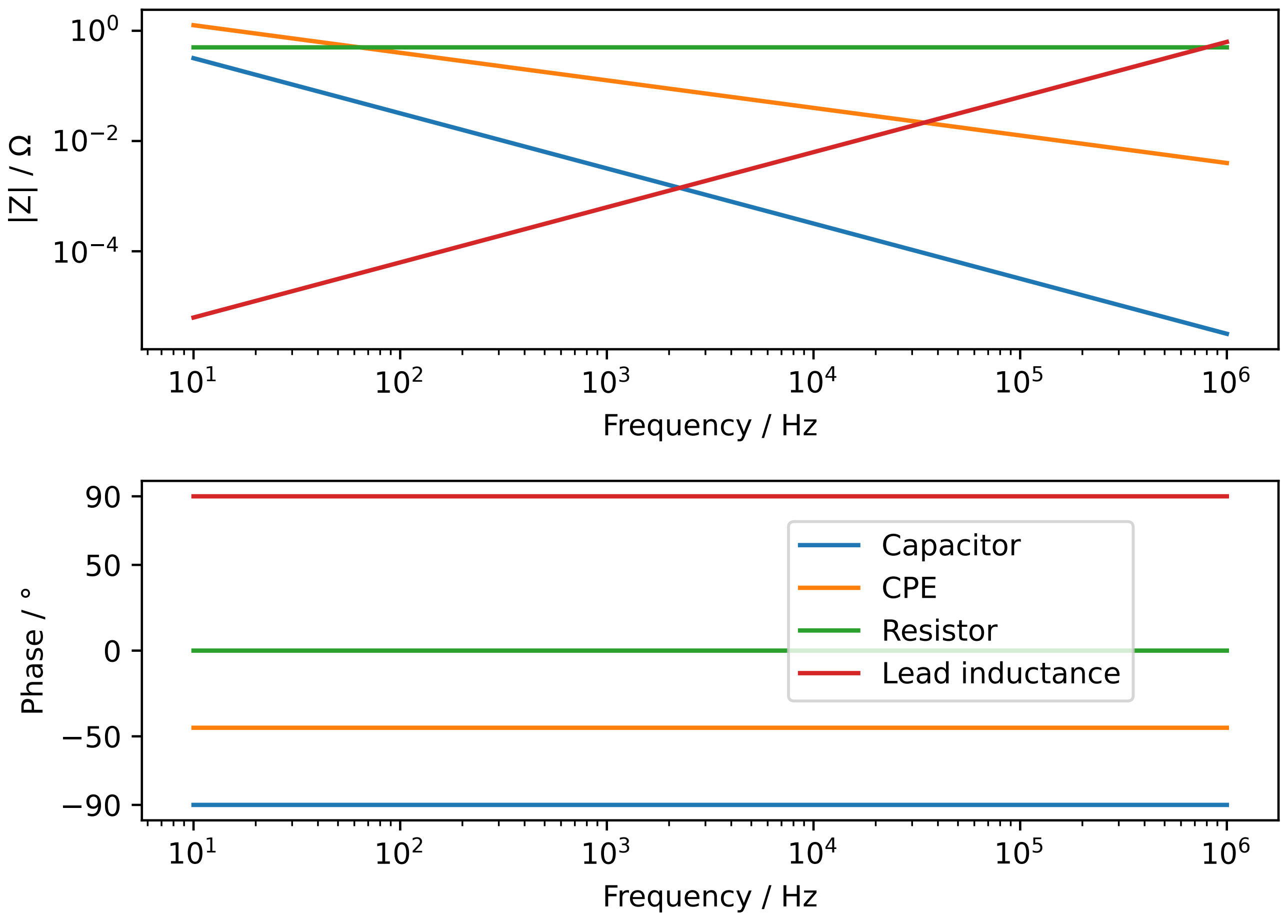

Figure S9: Bode plot of the impedance response of the basic elements of the circuit shown in Figure S7 for a capacitor of $50\,\mathrm{mF}$, a CPE (described by $\kappa(j\omega)^{-\alpha}$) with $\kappa = 10$ and $\alpha = 0.5$, a resistor of $0.5\,\Omega$ and a lead inductance of $100\,\mathrm{nH}$. The values were chosen arbitrarily. This figure is solely intended to highlight the characteristic appearance in Bode plot of the impedance response of the basic elements in the relevant frequency range. The capacitor has a fixed phase of $-90°$, the inductance has a fixed phase of $90°$, the resistor has a fixed phase of $0°$ and the CPE has a fixed phase of $-45°$, which is due to $\alpha = 0.5$. Aside from the resistor, all elements have frequency-dependent absolute values of the impedance. The impedance response of a real system may contain contributions from these elements, which vary over the measured frequency range. CPEs and capacitors influence mostly the low-frequency range (because the magnitude of their impedance decreases with increasing frequency), while inductances play only a role at higher frequencies (see, for example, Figure 6 of the manuscript).

4.1 Comparison of impedance spectra of KCl solutions

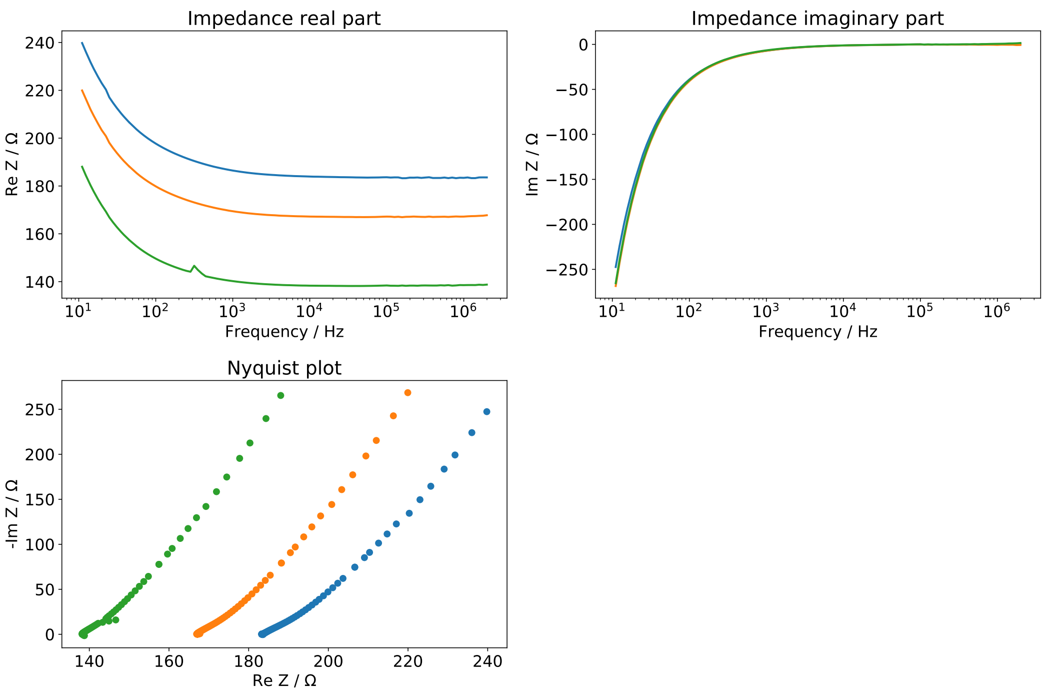

Figure S10: Impedance spectra of KCl solution with $3.5\,\mathrm{ml}$ (blue curves), $4\,\mathrm{ml}$ (orange curves), and $5\,\mathrm{ml}$ (green curves).

4.2 Nonlinear EIS

The impedance amplitude and phase at a fixed frequency of 130 Hz for increasing voltage amplitudes are shown in Figure S11.

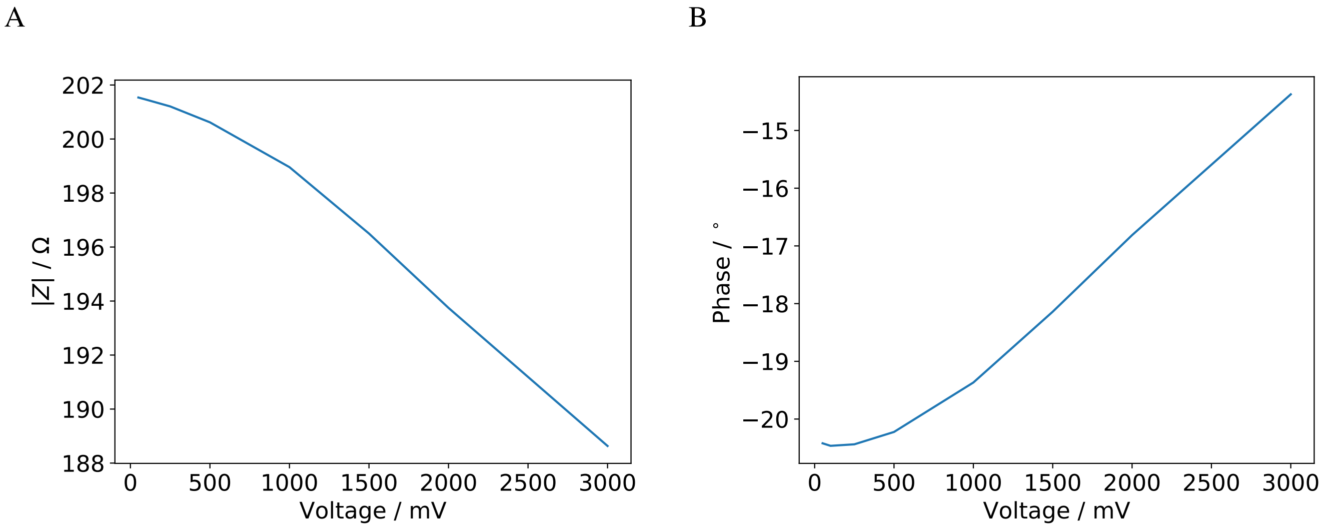

Figure S11: Impedance at 130 Hz for different voltage amplitudes. The impedance magnitude (**A**) and the phase (**B**) are shown.

5 NUMERICAL COMPUTATION OF STIMULATION WAVEFORMS OF RECTANGULAR TYPE

The complex-valued Fourier series of a signal f is given as

$$f(t) = \sum_{k=-\infty}^{\infty} c_k e^{jk\omega_0 t} \quad , \tag{S2}$$

where t is time, ω_0 the fundamental angular frequency and c_k are the complex-valued Fourier coefficients. The corresponding fundamental frequency is here denoted by f_0. For a monophasic rectangular pulse of amplitude A and pulse width t_p, the Fourier coefficients read

$$c_k = A t_p f_0 \operatorname{sinc}(k t_p f_0) \quad . \tag{S3}$$

Note that in this particular case, the coefficients c_k are real-valued. The amplitude of the frequency components A_k, which is relevant to estimate the strength of each harmonic, is given by

$$A_k = 2|c_k| \; \forall \; k > 0 \;\; \text{and} \;\; A_0 = c_0 \quad . \tag{S4}$$

The DC component (c_0) is only determined by the amplitude, pulse width and fundamental frequency

$$c_0 = A t_p f_0 \quad . \tag{S5}$$

In practice, the signal can be constructed using

$$f(t) = c_0 + 2\sum_{k=1}^{\infty} c_k e^{jk\omega_0 t} \tag{S6}$$

because the negative k-values would only affect the imaginary part, which for our application has no relevance.

Using this information, a biphasic rectangular pulse can be easily constructed as a superposition of two monophasic square waves. The square waves have to be time-shifted and their amplitudes need to have opposite signs. A time shift of the signal by t_s can be achieved by multiplying Equation (S3) by $e^{-jk\omega_0 t_s}$. For a biphasic pulse, the time shift has to be equal to the pulse width t_p. Writing the signals as a function $f(t, A, f_0, t_p, t_s)$, the signal of the biphasic rectangular pulse reads

$$f(t, A, f_0, t_p, 0) = f(t, A, f_0, t_p, 0) + f(t, -A, f_0, t_p, t_p) \quad . \tag{S7}$$

The amplitudes at the respective frequencies can then be obtained from Equation (S4). Similarly, a biphasic pulse with delay t_D can be constructed by

$$f(t, A, f_0, t_p, 0) = f(t, A, f_0, t_p, 0) + f(t, -A, f_0, t_p, t_p + t_D) \quad . \tag{S8}$$

Obviously, in both pulses the DC component cancels out because it only depends on amplitude and pulse width.

The presented approach is numerically very simple to implement and permits more flexibility than deriving the analytical presentation for each waveform. A drawback of the Fourier series approach are large

oscillations that occur at the jumps of the square waves (the so-called Gibbs phenomenon). We attenuated the oscillations using the Lanczos sigma factor in the summation (Weisstein, 2021). Furthermore, we used 2500 harmonics (i.e., including harmonics up to a frequency of $325\,\mathrm{kHz}$) to compute the signals unless otherwise stated. This covers the relevant part of the frequency spectrum for all considered signals (Figures S12–S14). Using this number of harmonics reduced oscillations caused by high-frequency contributions. This was only evident for current-controlled stimulation, where high-frequency oscillations occurred (Figures S15). They were most likely caused by the increased lead inductance. Such features were not measured. Reasons for that could be a low-pass filter of the stimulator or of the oscilloscope, which was used for recording. Importantly, the general signal shape of the predicted and measured signal agreed well.

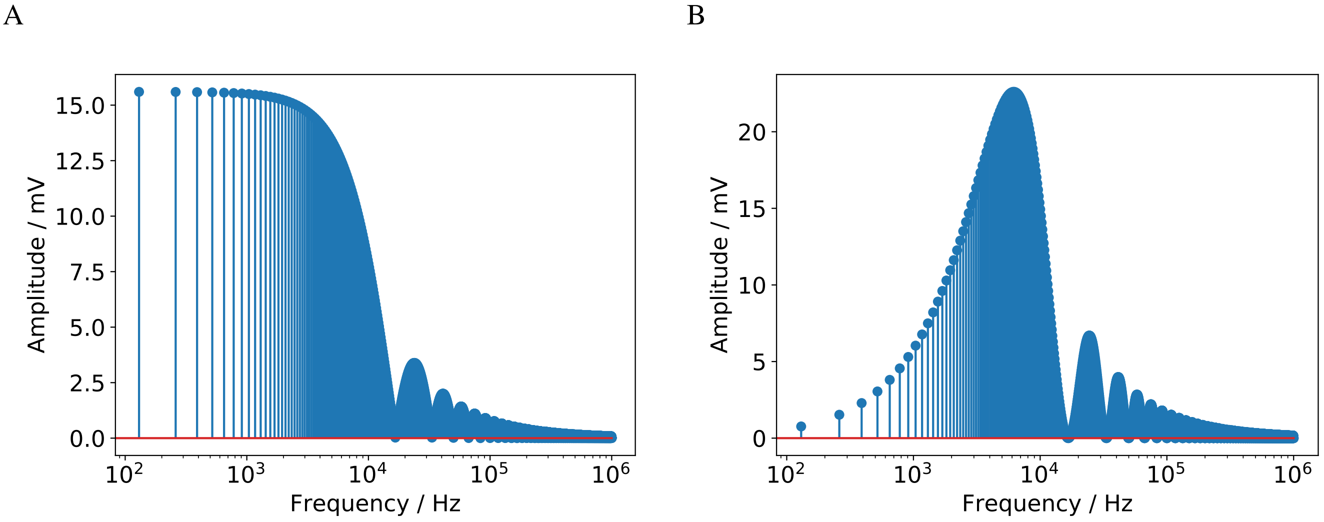

Figure S12: Fourier coefficients for a pulse width of $60\,\mu\mathrm{s}$. (**A**): monophasic pulse, (**B**): biphasic pulse. The DC component of the monophasic pulse is $7.8\,\mathrm{mV}$.

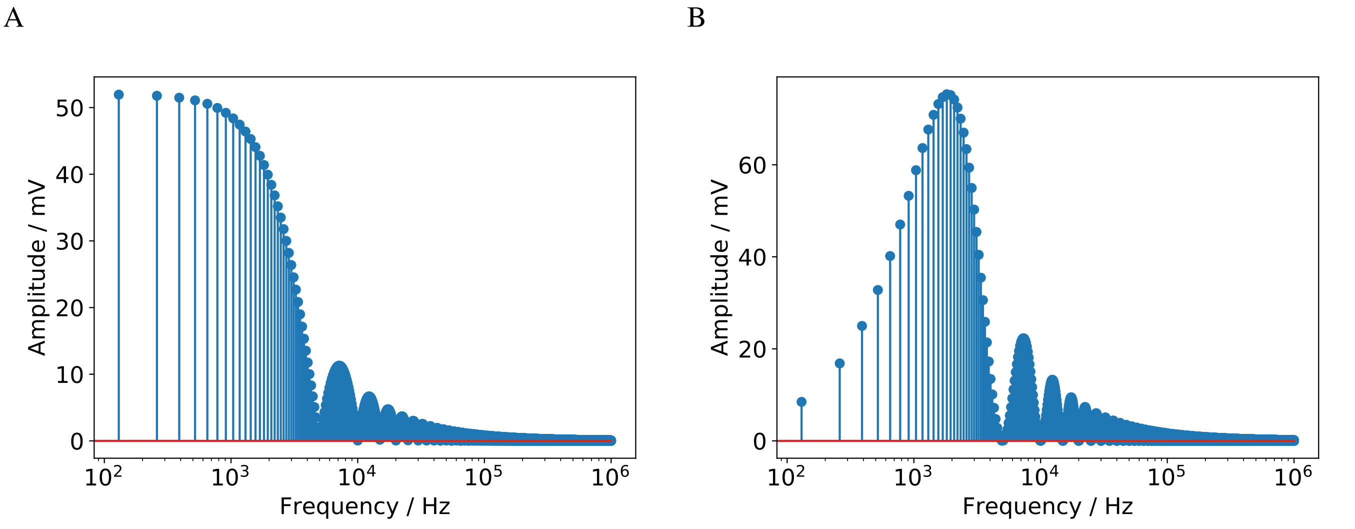

Figure S13: Fourier coefficients for a pulse width of $200\,\mu s$. (**A**): monophasic pulse, (**B**): biphasic pulse. The DC component of the monophasic pulse is $26\,\mathrm{mV}$.

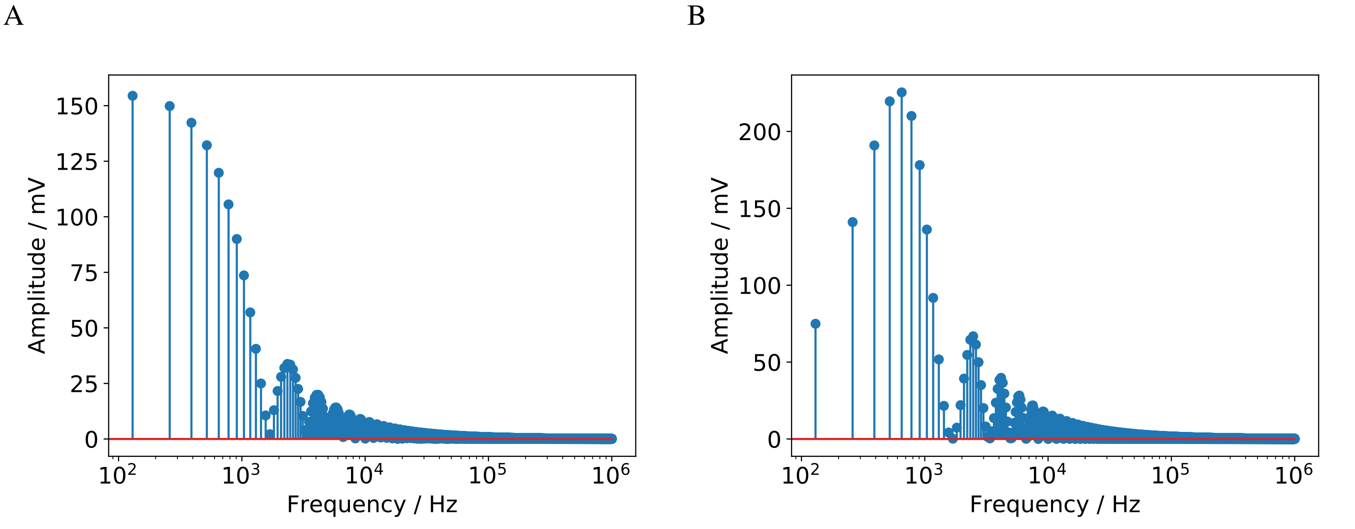

Figure S14: Fourier coefficients for a pulse width of $600\,\mu s$. (**A**): monophasic pulse, (**B**): biphasic pulse. The DC component of the monophasic pulse is $78\,\mathrm{mV}$.

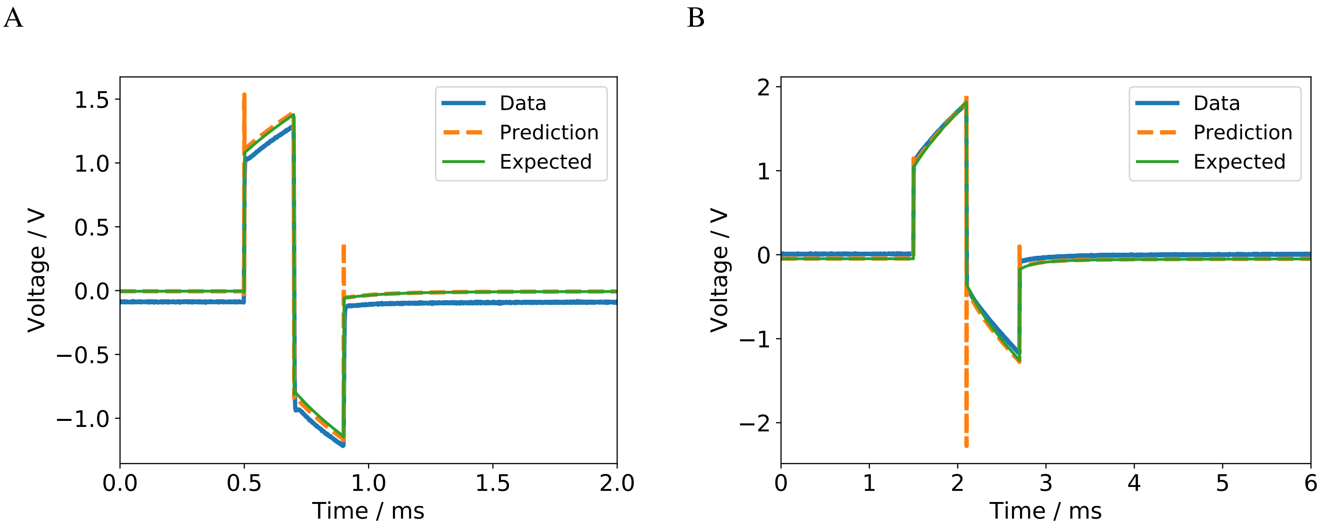

Figure S15: Voltage response for a biphasic pulse in current-controlled regime ($6.5\,\mathrm{mA}$ amplitude) with a pulse width of $200\,\mu\mathrm{s}$ (**A**) and $600\,\mu\mathrm{s}$ (**B**) for a single electrode pair. The experimental data is compared to the prediction based on the impedance model (computed using 5000 harmonics). For comparison, the expected voltage is shown: it was computed using the impedance measured by EIS and not inferred from the measured pulses. The overshooting in the prediction data arises due to the Fourier series approach and the high-frequency contribution of the lead inductance. It can be removed by using less harmonics (see Figure 8 in the manuscript, where 2500 harmonics were used while not changing the actual waveform).

5.1 Stimulation waveforms for particular settings

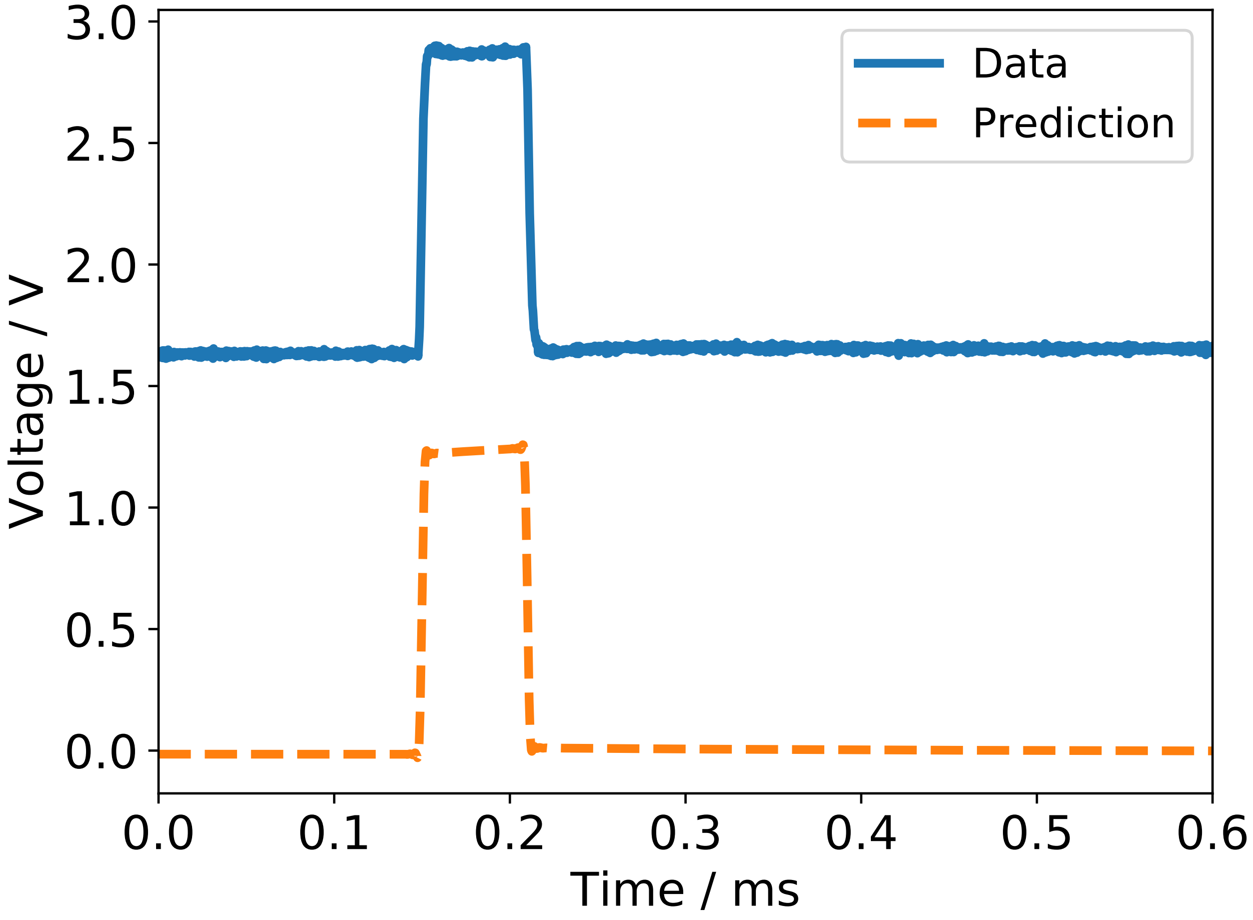

Figure S16: Measured and expected voltage transient for KCl solution with $3.5\,\mathrm{ml}$ stimulated with a current-controlled pulse ($6.5\,\mathrm{mA}$, $130\,\mathrm{Hz}$, $60\,\mu\mathrm{s}$).

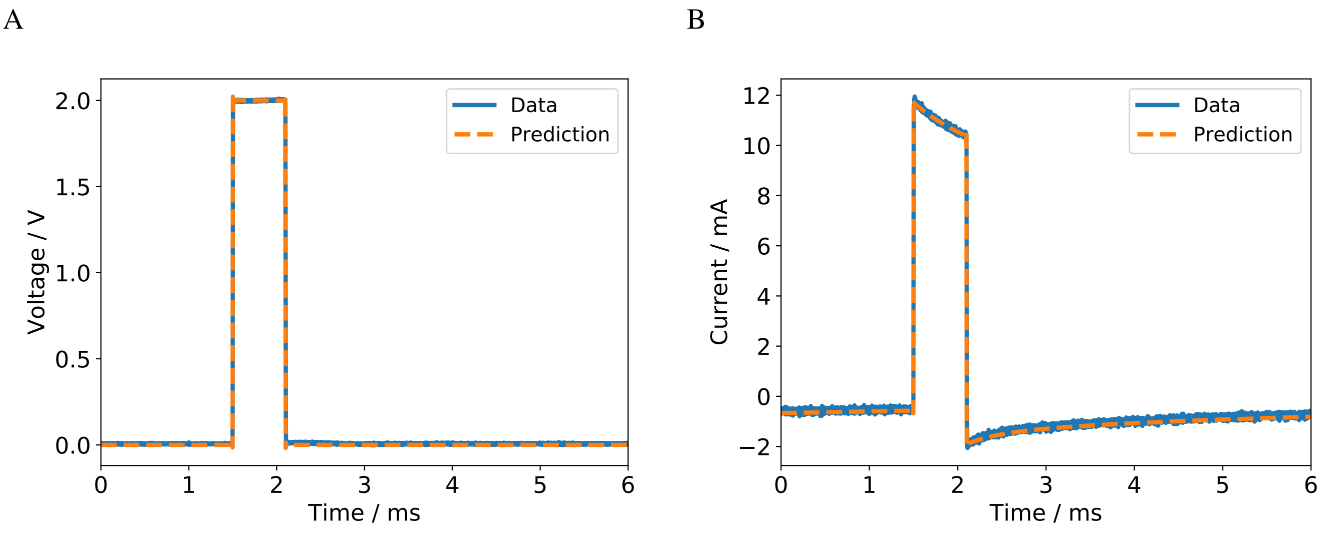

Figure S17: Measured and expected voltage (**A**) and current transient (**B**) for KCl solution with $4\,\mathrm{ml}$ stimulated with a voltage-controlled pulse ($600\,\mu\mathrm{s}$, $2\,\mathrm{V}$, $130\,\mathrm{Hz}$). A negative DC offset in the current is evident.

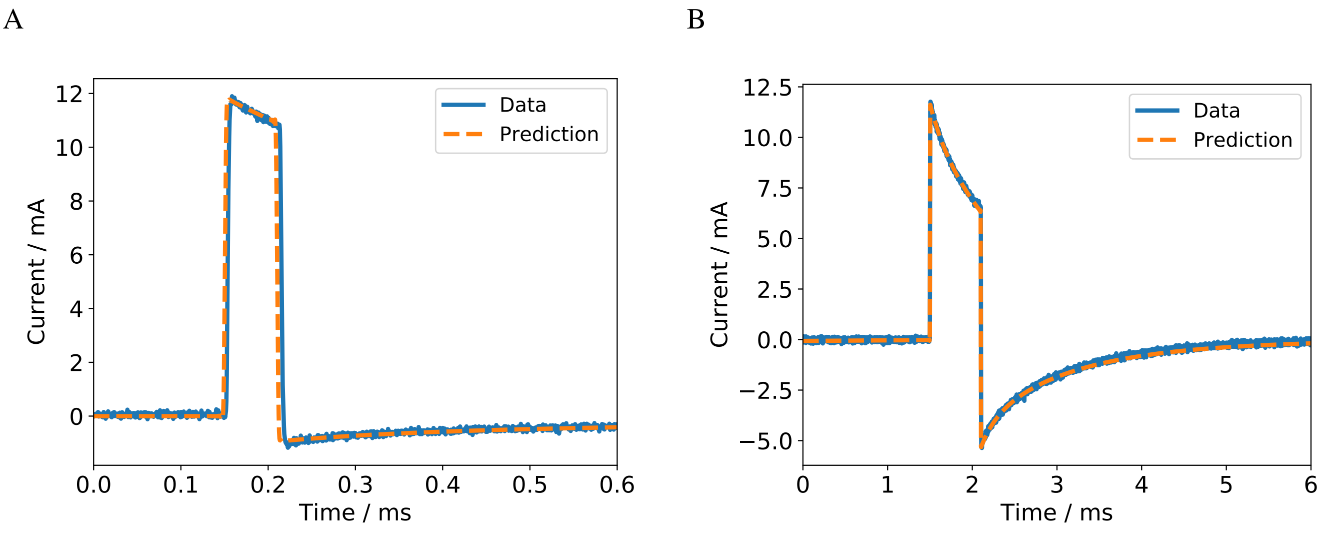

Figure S18: Current response for monophasic pulse in voltage-controlled regime ($2\,\mathrm{V}$ amplitude, $130\,\mathrm{Hz}$) with a pulse width of $60\,\mu\mathrm{s}$ (**A**) and $600\,\mu\mathrm{s}$ (**B**) for a single electrode pair. The experimental data is compared to the prediction based on the impedance model.

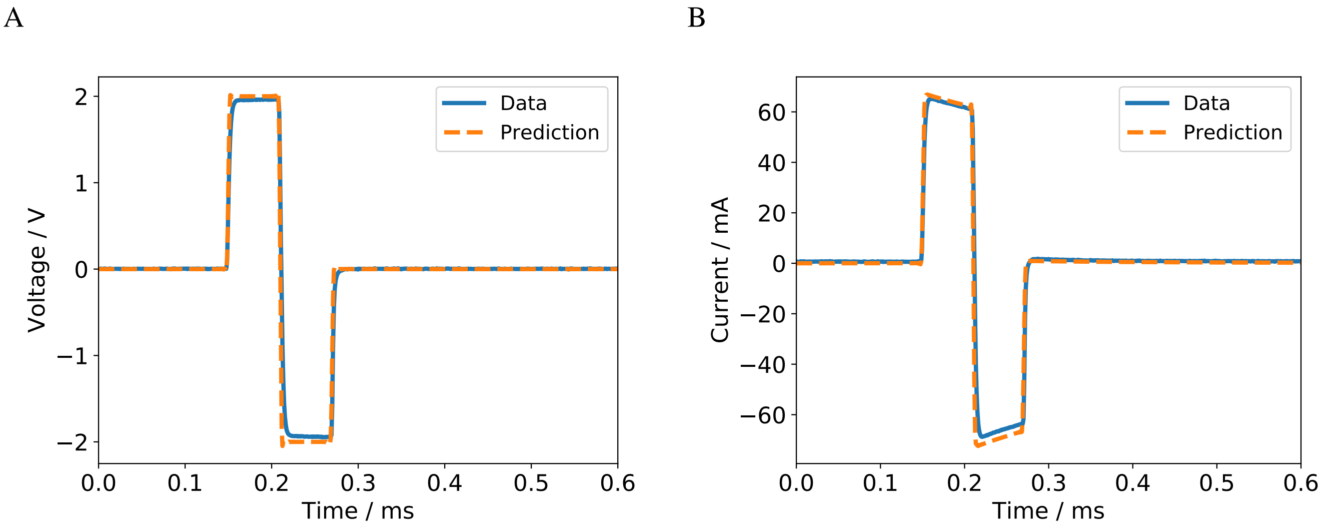

Figure S19: A biphasic pulse in voltage-controlled regime ($2\,\mathrm{V}$ amplitude, $130\,\mathrm{Hz}$) with a pulse width of $60\,\mu\mathrm{s}$ (**A**) was applied on six wells connected in parallel. In comparison to the single-well configuration (Fig. S17), the supplied voltage waveform deviates from the expected waveform (**A**). The resulting current is shown in the right panel (**B**). The experimental data is compared to the prediction based on the impedance model. A slight DC offset of unknown origin is evident in the right panel, which indicates DC contamination of the current.

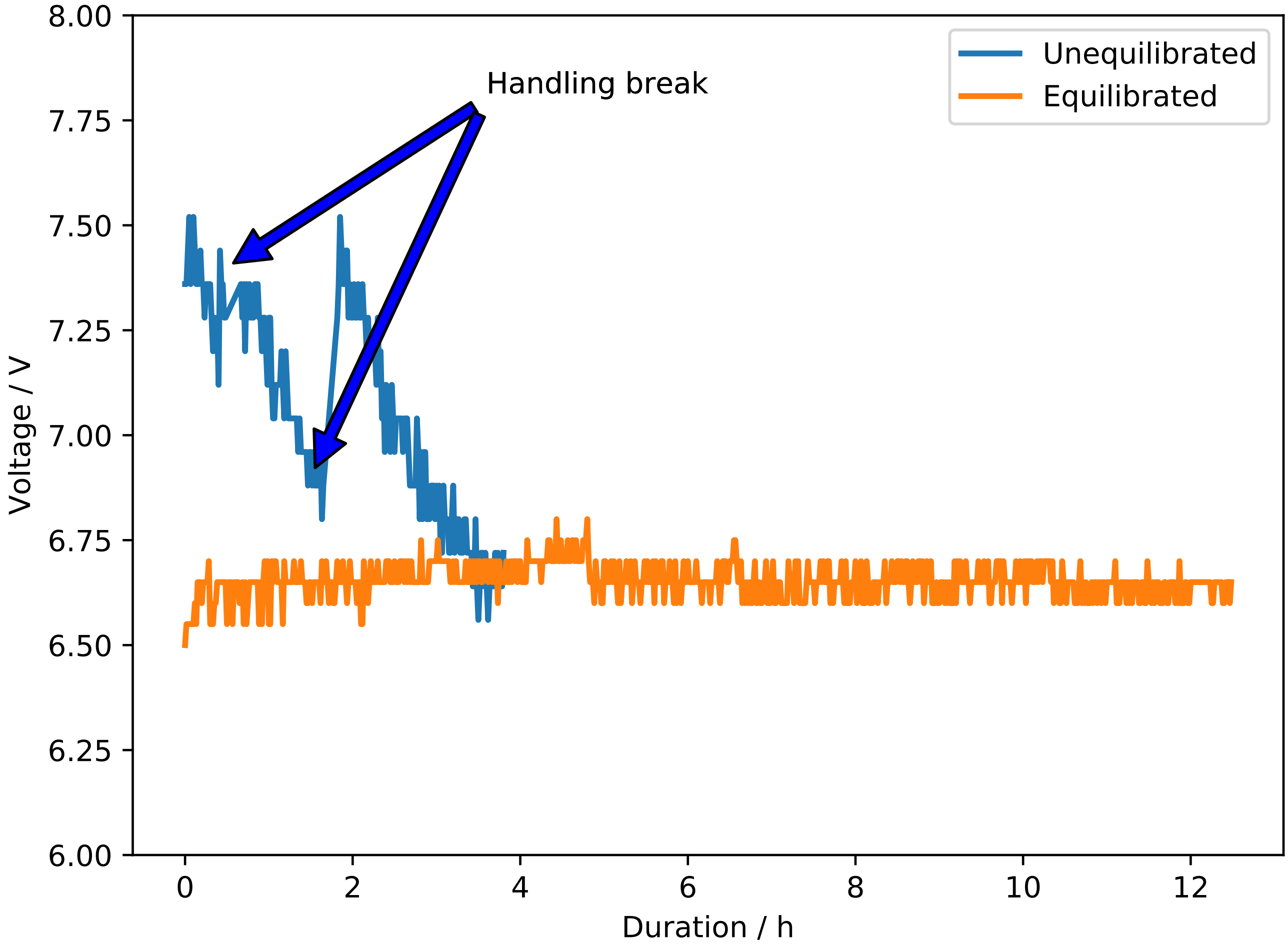

Figure S20: Comparison of stimulation voltages (peak-to-peak) for current-controlled stimulation ($6.5\,\mathrm{mA}$ amplitude, $130\,\mathrm{Hz}$, $60\,\mu\mathrm{s}$) when the system was used directly after handling without thermal equilibration (blue) or after thermal equilibration took place (i.e., at $37\,^{\circ}\mathrm{C}$) (orange). The stimulation was performed with three (out of six) filled wells in series (without thermal equilibration) or six filled wells (with thermal equilibration). For the sake of comparison, the results for the thermally equilibrated system were divided by two to account for the additional wells. Because we could show that the wells are equal from an electrochemical point of view, this approach is feasible and does not introduce an error. When the thermally unequilibrated system was used, three 6-well plates, containing fresh cell culture medium, were stimulated successively either for 30 minutes, 60 minutes, or 120 minutes including short handling breaks in between. During these handling breaks, the lid was cleaned and kept at room temperature and the ambient temperature in the incubator dropped by a few degrees Celsius. The stimulation of the thermally unequilibrated system was completed after 4 hours. The stimulation of the thermally equilibrated system was continuously applied for 12 hours.

5.2 Comparison of lumped and distributed impedance model

In this work, we estimated the electric field in the medium by the current-conductivity and the voltage-divider method. Both methods rely on a lumped-element model (i.e., the equivalent circuit model) that makes the assumption that the EEI impedance does not affect the voltage distribution in the medium. For Dirichlet boundary conditions, this means that we assumed that the voltage in the medium is constant at the electrode surface. Another class of boundary conditions, Robin or mixed boundary conditions, permits to model a distributed impedance. Then, the EEI impedance is, for example, assumed to be equally distributed on the electrode surface. The specific impedance of the electrode surface is $z = \frac{Z}{A}$, where Z is the (lumped) EEI impedance and A is the electrode surface area. The Robin boundary condition, which is imposed instead of the Dirichlet boundary condition, is formulated for the normal component of the current density J_n and reads

$$-J_n = \frac{1}{z}(\Phi - \Phi_{\mathrm{ref}}) \ . \tag{S9}$$

Here, Φ_{ref} is the stimulation voltage and Φ the unknown potential to be computed. Note that Φ_{ref} is usually by the Dirichlet boundary condition in the lumped-element model. In Figure 10 in the manuscript, we compare three possible modelling assumptions and their results for the model geometry, which we used in Sec. 2 of the manuscript to verify our implementation. As already concluded in other studies (Howell et al., 2014), for homogeneous fields as considered here the results obtained by the different approaches do not differ significantly. However, it could be that the error of the field strength estimated by the UQ approach underestimates the error by about 1% or 2%.

5.3 Estimation of temperature change in medium after adding electrodes

Using the following parameter values:

- $c_{\text{Pt}} = 130\,\text{J}\,\text{kg}^{-1}\,\text{K}^{-1}$
- $c_{\text{H}_2\text{O}} = 4185\,\text{J}\,\text{kg}^{-1}\,\text{K}^{-1}$
- $m_{\text{Pt}} = \rho_{\text{Pt}} V_{\text{Pt}} = \rho_{\text{Pt}} \cdot 2 \cdot A \cdot l = 21.45\,\text{g/cm}^3 \cdot 2 \cdot \pi \cdot (0.5 \times 10^{-1}\,\text{cm})^2 \cdot 4\,\text{cm} = 1.348 \times 10^{-3}\,\text{kg}$
- $m_{\text{H}_2\text{O}} = \rho_{\text{H}_2\text{O}} V_{\text{H}_2\text{O}} = 1\,\text{g/cm}^3 \cdot 4\,\text{mL} = 4 \times 10^{-3}\,\text{kg}$
- $T_{\text{hot}} = 310\,\text{K}$, $T_{\text{cold}} = 283\,\text{K}$

we estimated the mixture temperature and subtracted it from the incubator temperature T_{hot}

For this, consider that the equilibrium of the thermal energies Q

$$
\begin{aligned}
Q_{\text{in}} &= -Q_{\text{out}} \\
c_{\text{Pt}} m_{\text{Pt}} \Delta T_{\text{Pt}} &= -c_{\text{H}_2\text{O}} m_{\text{H}_2\text{O}} \Delta T_{\text{H}_2\text{O}} \\
c_{\text{Pt}} m_{\text{Pt}} (T_{\text{mix}} - T_{\text{cold}}) &= -c_{\text{H}_2\text{O}} m_{\text{H}_2\text{O}} (T_{\text{mix}} - T_{\text{hot}}) \\
T_{\text{mix}} &= \frac{c_{\text{H}_2\text{O}} m_{\text{H}_2\text{O}} T_{\text{hot}} + c_{\text{Pt}} m_{\text{Pt}} T_{\text{cold}}}{c_{\text{H}_2\text{O}} m_{\text{H}_2\text{O}} + c_{\text{Pt}} m_{\text{Pt}}} \quad ,
\end{aligned}
$$

which yields

$$
\begin{aligned}
\Delta T &= T_{\text{hot}} - T_{\text{mix}} \\
&= 310\,\text{K} - \frac{4185\,\text{J}\,\text{kg}^{-1}\,\text{K}^{-1} \cdot 4 \times 10^{-3}\,\text{kg} \cdot 310\,\text{K} + 130\,\text{J}\,\text{kg}^{-1}\,\text{K}^{-1} \cdot 1.348 \times 10^{-3}\,\text{kg} \cdot 283\,\text{K}}{4185\,\text{J}\,\text{kg}^{-1}\,\text{K}^{-1} \cdot 4 \times 10^{-3}\,\text{kg} + 130\,\text{J}\,\text{kg}^{-1}\,\text{K}^{-1} \cdot 1.348 \times 10^{-3}\,\text{kg}} \\
\Delta T &\approx 0.3\,\text{K}
\end{aligned}
$$

5.4 Provenance graph showing all experiments done for this publication as well as the development of the digital twin of the stimulation chamber

Figure S21 displays the provenance graph applying the PROV Data Model (Belhajjame et al., 2013) showing all experimental and modelling steps needed for a digital twin of the stimulation device. The graph comprises entities (round shape) as well as activities (rectangular shape). The relations (i.e., *used* and *generated by*) are marked by arrows showing backwards in time. Activities *use* entities and entities are generated by activities.

We have highlighted the three activities that are featured in our work: prior (electrochemical) characterisation of the stimulation device, *in silico* modelling and analysis of the models and measurement data, as well as *in situ* measurements needed for applying the digital twin.

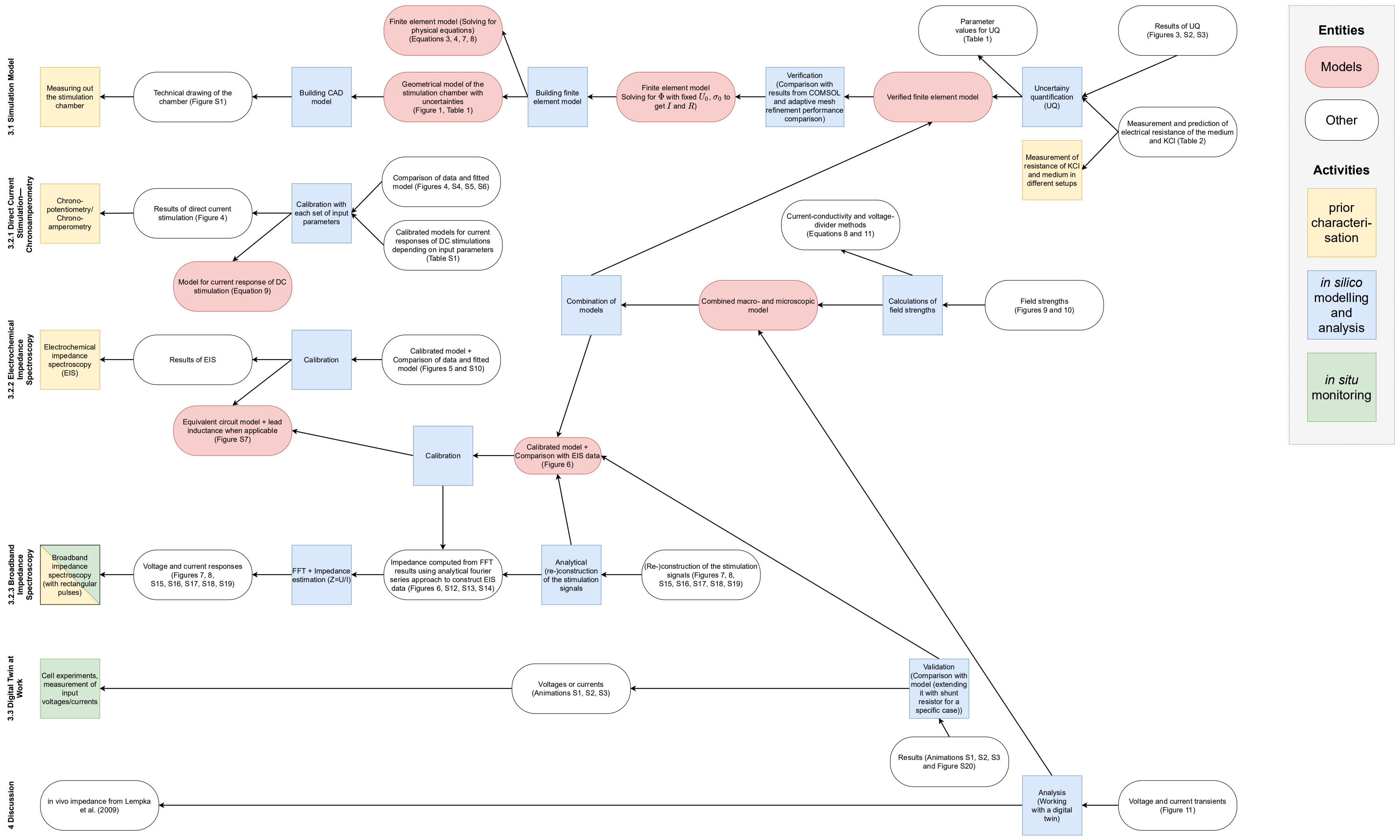

Figure S21: Provenance graph showing entites and activities. We show three major activities as well as the entity “Model”. The relations (i.e., arrows) indicate which entities are being generated or used by which activities.

REFERENCES

Bard, A. J. and Faulkner, L. R. (2001). *Electrochemical methods : fundamentals and applications* (New York: Wiley), 2. ed. edn.

Belhajjame, K., B'Far, R., Cheney, J., Coppens, S., Cresswell, S., Gil, Y., et al. (2013). *Prov-DM: The prov data model*. W3c recommendation, World Wide Web Consortium (W3C). Https://www.w3.org/TR/2013/REC-prov-dm-20130430/

Howell, B., Naik, S., and Grill, W. M. (2014). Influences of interpolation error, electrode geometry, and the electrode-tissue interface on models of electric fields produced by deep brain stimulation. *IEEE Trans. Biomed. Eng.* 61, 297–307. doi:10.1109/TBME.2013.2292025

Ragoisha, G. A., Osipovich, N. P., Bondarenko, A. S., Zhang, J., Kocha, S., and Iiyama, A. (2010). Characterisation of the electrochemical redox behaviour of Pt electrodes by potentiodynamic electrochemical impedance spectroscopy. *J. Solid State Electrochem.* 14, 531–542. doi:10.1007/s10008-008-0663-7

[Dataset] Weisstein, E. W. (2021). Lanczos sigma factor. From MathWorld—A Wolfram Web Resource. Last visited on 29/05/2021
